# Resolution-Phase Immunometabolic Reprogramming by *Gelidiopsis variabilis* Polysaccharides: Macrophage Polarization and *In Vivo* Anti-Inflammatory Efficacy

**DOI:** 10.3390/md24090299

**Published:** 2026-08-27

**Authors:** Amal D. Premarathna, Katarzyna M. Dziubinska-Kuehn, Muthupandian Saravanan, Anti Sooäär, Indrek Reile, Tamer A. E. Ahmed, Maxwell T. Hincke, Rando Tuvikene

**Affiliations:** 1 School of Natural Sciences and Health, Tallinn University, Narva mnt 29, 10120 Tallinn, Estonia; antisoo22r@gmail.com (A.S.); rantuv@tlu.ee (R.T.); 2 National Institute of Chemical Physics and Biophysics, Akadeemia tee 23, 12618 Tallinn, Estonia; dziub.kat@gmail.com (K.M.D.-K.); indrek.reile@kbfi.ee (I.R.); 3 Prince Fahad bin Sultan Chair for Biomedical Research, University of Tabuk, Tabuk 71491, Saudi Arabia; smuthupandian@ut.edu.sa; 4 Department of Medical Laboratory Technology, Faculty of Applied Medical Sciences, University of Tabuk, Tabuk 71491, Saudi Arabia; 5 Department of Cellular and Molecular Medicine, Faculty of Medicine, University of Ottawa, Ottawa, ON K1H 8M5, Canada; tahmed@uottawa.ca (T.A.E.A.); mhincke@uottawa.ca (M.T.H.); 6 Department of Innovation in Medical Education, Faculty of Medicine, University of Ottawa, Ottawa, ON K1H 8M5, Canada

**Keywords:** *Gelidiopsis variabilis*, funoran, immunometabolic reprogramming, macrophage polarization, resolution-phase inflammation, anti-inflammatory

## Abstract

Sulfated polysaccharides from red seaweeds are emerging as versatile bioactive macromolecules. Here, we report that funoran-type galactans from *Gelidiopsis variabilis* (GV) function as immunometabolic reprogrammers, orchestrating inflammation resolution through targeted remodeling of amino acid and fatty acid metabolism in macrophages, potentially via receptor-mediated pathways. Cold and hot water extraction yielded structurally distinct fractions (360–3006 kDa) characterized by NMR as sulfated galactans with κ-carrageenan motifs and variable sulfation (6.6–21.5%). In RAW264.7 macrophages, fractions GV-1A and GV-2A induced an anti-inflammatory metabolic state characterized by arginine accumulation (from 4.61% to 7.06% of the total amino acid pool), reduced ornithine levels, and complete the elimination of pro-inflammatory myristate (14:0), while upregulating phagocytosis (149% of control). In a carrageenan-induced paw edema model, GV-1A and GV-2A dose-dependently upregulated pro-resolution markers (*IL-10*, *IL-4*, *TGF-β1*, *Arg1*, HO-1), outperforming the standard drug meloxicam. While the *in vitro* arginine accumulation suggests functional arginase inhibition, the *in vivo Arg1* upregulation reflects a transcriptional response in a complex tissue environment. We propose that these findings collectively support a ‘resolution-phase metabolic phenotype’, a working model whereby GV polysaccharides reprogram macrophage metabolism toward inflammation resolution through context-dependent mechanisms. These findings establish *G. variabilis* polysaccharides as metabolically active immunomodulators that promote inflammation resolution, with the potential for applications in inflammatory diseases and as a pharmaceutical platform for drug development.

## 1. Introduction

Red algae (Rhodophyta) are a rich source of sulfated galactans (agar, carrageenan, funoran) with tunable properties and broad bioactivities [1,2]. However, unlike agar and carrageenan, funoran remains underexplored, particularly regarding structure–function relationships and immune cell-specific effects. Traditionally isolated from *Gloiopeltis*, funoran is characterized by repeating G6S- la diads (β-d-galactose-6-sulfate linked to 3,6-anhydro-α-l-galactose) [3,4]. Variations in sulfation, molecular weight, and substitution drive structural heterogeneity and reported bioactivities (anticoagulant, antioxidant, anti-inflammatory, immunomodulatory, etc.). *Gelidiopsis* spp. are red seaweeds from tropical/subtropical waters, primarily studied for their funoran-type sulfated galactans [5]. GV-derived funoran is predicted to differ structurally from *Gloiopeltis* due to divergent phylogenetic and ecological contexts, yet no systematic study has investigated *Gelidiopsis variabilis* (GV) funoran. This represents a critical knowledge gap, both taxonomically (GV has not been characterized) and mechanistically (the immunomodulatory mechanisms of funorans remain poorly defined).

While marine algal polysaccharides are increasingly recognized for immune modulation [6,7], the mechanisms remain incompletely understood. Most studies attribute bioactivity to charge-dependent receptor engagement (e.g., TLR4, dectin-1, CR3) and downstream NF-κB or MAPK signaling [8]. However, emerging evidence suggests that polysaccharides can induce deeper metabolic changes in immune cells, including altered amino acid metabolism, lipid remodeling, and shifts in oxidative phosphorylation [9]. These observations raise the possibility that polysaccharides may function as immunometabolic modulators rather than merely surface receptor agonists.

We hypothesize that GV funorans act as potential immunometabolic modulators, reshaping amino acid and fatty acid flux in macrophages in a manner associated with inflammation resolution. This contrasts with the prevailing view that sulfated galactans act primarily via receptor binding and challenges the field to consider metabolic mechanisms. Specifically, we predict that (1) cold-extracted, low-MW fractions (GV-1A) induce a pro-resolution macrophage polarization via arginine accumulation and myristate elimination; (2) these fractions upregulate resolution-phase markers (*Arg1*, *IL-10*, HO-1) *in vivo*; and (3) GV fractions will outperform conventional NSAIDs by promoting transcriptional changes consistent with resolution rather than merely suppressing inflammation.

To evaluate these predictions experimentally, we isolated GV polysaccharides using sequential cold/hot extraction, characterized them via HP-SEC, FTIR, and NMR, and evaluated immunomodulation in RAW264.7 macrophages with integrated amino acid and fatty acid metabolomics. By combining structural characterization with functional assays and metabolomics, this study moves beyond descriptive bioactivity to establish causation, demonstrating that GV funorans reprogram macrophage metabolism. This work addresses a critical gap in funoran research and establishes GV polysaccharides as metabolically active anti-inflammatory biomaterials with therapeutic potential for inflammatory diseases, positioning them as candidates for future resolution-based therapeutics.

## 2. Results and Discussion

### 2.1. Physical and Chemical Properties of the Extracted Funoran

#### 2.1.1. Extraction Yield, Molecular Weights

**Figure 1:**
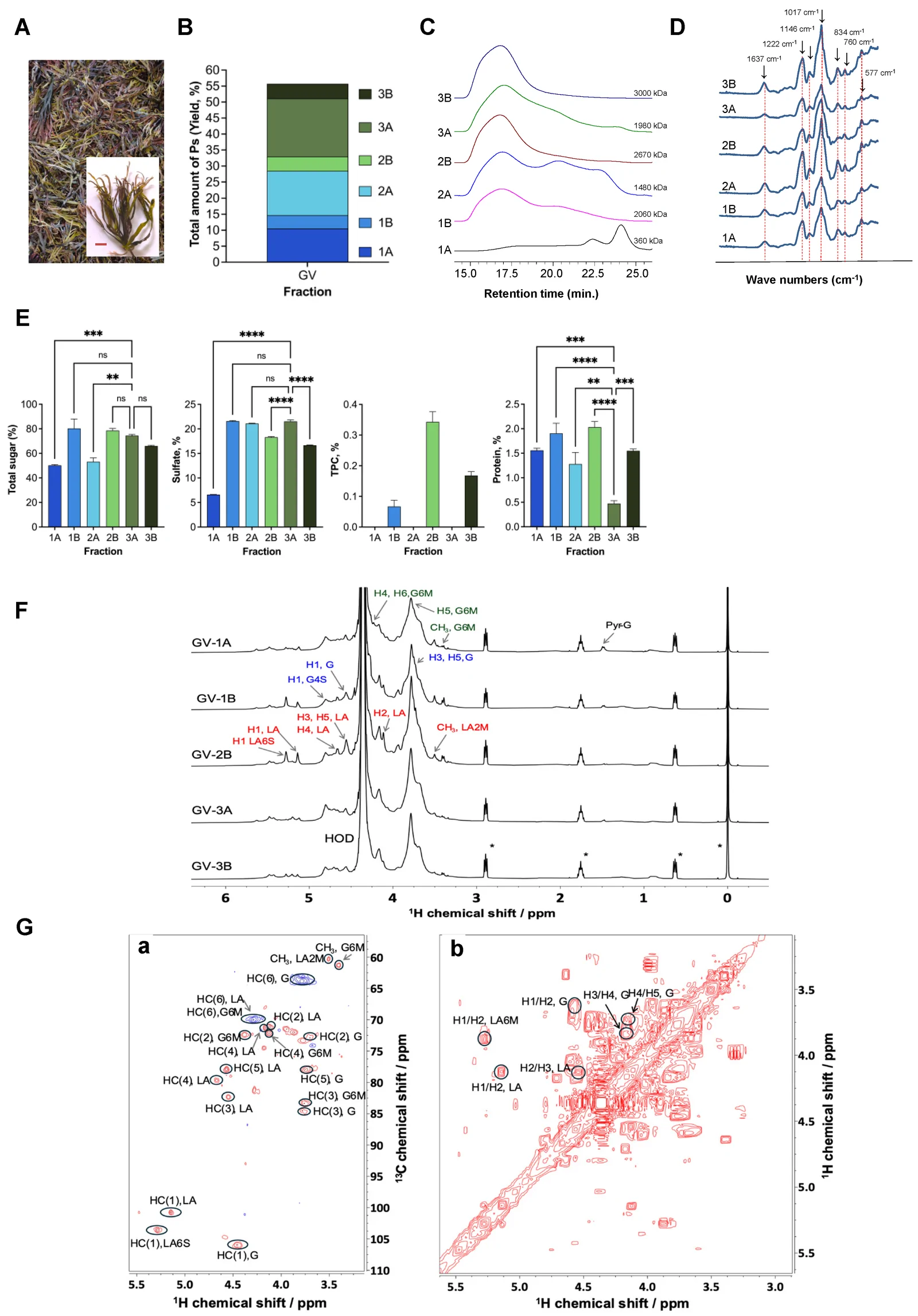
(**A**). Red seaweed—dried biomass: *G. variabilis* (GV). Scale bar: 1 cm. (**B**). Total amount of polysaccharides (%) from each GV fraction. (**C**). Size-exclusion chromatography elution profiles comparing funoran fractions. (**D**). FT-IR spectra of red seaweed polysaccharide samples; the IR spectra within the 500–2000 cm−1 wavenumber region (fingerprint region for polysaccharides) were used for data analyses. (**E**). Chemical Composition (Sulfate, TPC, Protein, and Total Sugar) of GV Fractions. Data were expressed as mean ± SEM (*n* = 4); data are compared to values of fraction 3A. **** *p* < 0.0001, *** *p* < 0.001, and ** *p* < 0.01were considered statistically significant compared to the control group. (*) indicates a statistically significant difference using ANOVA, followed by Dunnett comparisons test. (**F**). ^1^H NMR spectra of sulfated funoran-type galactans from *G. variabilis* (fractions 1A, 1B, 2B, 3A and 3B). All spectra were acquired at 65 °C. The three most populated species in these samples are β-d-galactose (G, blue), 6-methylated β-d-galactose (G6M, green), and 3,6-anhydro-α-l-galactose (la, red). The asterisk (*) indicates the DSS signal. (**G**). ^1^H-^13^C 2D HSQC NMR spectra in D_2_O at 65 °C. panel (**a**): Fraction GV-1B. Characteristic ^1^H/^13^C correlations for β-d-galactose (G), 6-methylated β-d-galactose (G6M), and 3,6-anhydro-α-l-galactose (la) are labeled. Panel (**b**): Fraction GV-1B. Identified 1H/1H correlations in G, G6M and la are labeled. The summary of all chemical shifts in G, G6M, and la is presented in Table 1.

The polysaccharide fractions isolated from *G. variabilis* (GV) demonstrated a total extraction yield of 55.7% of the dry biomass, with recovery efficiency strongly dependent on extraction parameters (temperature, extraction time, etc.). Cold-water extraction at 25 °C yielded comparatively lower polysaccharide amounts (GV-1A: 10.5%; GV-1B: 4.2%; total cold-extracted: 14.7%), while hot-water extraction at 95 °C significantly enhanced recovery across all fractions (GV-2A: 13.8%; GV-2B: 4.5%; GV-3A: 18.1%; GV-3B: 4.6%; total hot-extracted: 41.0%). GV-3A emerged as the most productive single fraction (18.1% yield; Figure 1B).

Molecular characterization revealed substantial variations in weight-average molecular weights (MW), ranging from 360 kDa (GV-1A) to 3006 kDa (GV-3B). The cold-extracted fractions exhibited particularly broad molecular weight distributions (GV-1A: 360 kDa; GV-1B: 2060 kDa), whereas hot-extracted fractions consistently produced higher weight-average molecular weight polymers (GV-2A: 1480 kDa; GV-2B: 2670 kDa; GV-3A: 1980 kDa; GV-3B: 3000 kDa) (Table S1, Figure 1C). This molecular weight stratification correlates with extraction temperature, where elevated temperatures appear to preferentially solubilize larger polysaccharide assemblies [10]. The polydispersity indices followed a parallel trend, with cold fractions demonstrating broader molecular weight distributions compared to the more homogeneous hot-extracted fractions.

#### 2.1.2. Fourier-Transform Infrared Spectroscopy

The structural features of all six GV polysaccharide fractions (GV-1A, 1B, 2A, 2B, 3A, 3B) were elucidated by FTIR spectroscopy. Characteristic absorption patterns consistent with sulfated galactan structures were detected (Figure 1D). A broad band at 3392–3398 cm^−1^ was attributed to O-H stretching, indicative of the extensive hydrogen-bonding network typical of polysaccharides [11]. Most fractions showed minimal absorption in the 2900 cm^−1^ region, whereas GV-1B, 2B, and 3B displayed minor signals at approximately 2920 cm^−1^, corresponding to C-H stretching. Diagnostically important absorptions were identified in the mid-infrared region: a band at 1646–1636 cm^−1^ (bound water or C=C stretching), strong signals at 1221–1223 cm^−1^ and 1144–1149 cm^−1^ (asymmetric S=O and C-O-C glycosidic bridge stretching), and a distinct absorption at 1016–1018 cm^−1^ (symmetric S=O stretching of sulfate esters), confirming the sulfated nature of these galactans [12]. The fingerprint region revealed vibrations at 834–838 cm^−1^ (C-O-S bending of sulfate), 758–764 cm^−1^ (C-C bending), and 575–577 cm^−1^ (C-H deformation of galactose residues). Although characteristic IR absorptions for 3,6-anhydrogalactose (~1070 and 933 cm^−1^) were not clearly observed, NMR spectroscopy confirmed their presence. The sulfate substitution pattern was elucidated by diagnostic bands at 822 cm^−1^ (galactose-6-sulfate, G6S) and 870 cm^−1^ (6-O-sulfated anhydrogalactose, L6S), indicating both major sulfated units [13]. The most intense IR peak was a single, symmetrical peak centered at 1068 cm^−1^ (1030–1110 cm^−1^), representing overlapping C-O/C-C stretching and deformation modes. This spectral signature, distinctly different from the doublet pattern characteristic of agarose [14], serves as a definitive marker for identifying these extracts as funoran-type sulfated galactans.

#### 2.1.3. NMR Analysis—^1^H and ^13^C-NMR Spectra

The chemical structure of *G. variabilis* polysaccharides was investigated using NMR spectroscopy. The 1D and 2D NMR analyses confirmed the presence of monomers and dimers characteristic of the funoran units, also observed in the FTIR spectra. Overall, four species could be confidently assigned based on the detected ^1^H/^13^C (HSQC) and 1H/1H (COSY) correlations: β-d-galactose (G), 6-methylated-β-d-galactose (G6M), β-d-galactose-6-sulfate (G6S), and 3,6-anhydro-l-galactose (la). These assignments were further supported by the presence of patterns typical for methoxyl (-OCH3), sulfate (-SO3), and acetyl (-OCOCH3) substitutions. A summary of the ^1^H and ^13^C chemical shifts (CS) for the main species, determined in exemplary fraction GV-2A, is available in Table 1.

Figure 1F shows a comparison of ^1^H 1D NMR spectra measured across all solutions. Three of the detected polysaccharide fragments were labeled based on their relative contributions to different fractions: β-d-galactose (G, blue) in GV-1B, 6-methylated-β-d-galactose (G6M, green) in GV-1A, and 3,6-anhydro- l-galactose (la, red) in GV-2B. The resonances characteristic of the anomeric protons in la and 4-O-linked 6-sulfated-α-L-galactose (L6S) were observed at ^1^H/^13^C 5.12/100.8 ppm and 5.29/103.4 ppm, respectively [4]. The anomeric protons in β-D-galactose were assigned at 4.58/104.8 ppm, and anomeric protons of 4-sulfated-d-galactose (G4S) and G6M were observed at 4.46/106.01 and 4.80/103.6 ppm, respectively [3].

In all fractions, ^1^H NMR spectra showed the presence of methoxyl diads, particularly the diagnostic signal of a methyl group attached to C2 in LA2M at 3.50/60.3 ppm or C6 in G6M at 3.41/61.3 ppm. Salt precipitation introduced significant structural variations, with salt-precipitated fractions 1B and 2B exhibiting relatively constant LA2M content but lower G6M abundance compared to their non-salt-precipitated counterparts in 1A. In fractions 3A and 3B, this trend was not observed. Next, a comparative analysis of extraction methods demonstrated that successive hot- and cold-water extractions resulted in a gradual reduction in methoxyl group content, usually observed at 0.92/20.6 ppm in galactans. Sulfate precursor units (G6M and LA6M) were identified through the identification of multiple resonances, including not only the aforementioned anomeric protons, but also the following ring correlations in G6M: H2/C2 at 4.30/72.3 ppm; H3/C3 at 3.80/82.1 ppm; H4/C4 at 4.20/71.1 ppm and characteristic H6/C6 at 4.20/69.6 ppm [15].

As seen in Figure 1F (^1^H comparison), the highest abundance of LA was observed in fraction GV-2B, slightly decreased in 1B, and significantly lower in 1A, 3A, and 3B. In parallel, the highest content of G was in 2B, and the other four fractions revealed a lower, but also comparable, concentration of the main galactan unit. The sulfated precursors, LA6S and G6M, showed particularly high abundance in 1A, 1B, and 2B, and were not observed in 3B or 3A, respectively. Fraction 1A also contained significantly more LA6M than G6M. The spectra also displayed minor signals from unsulfated G-LA2M diads and agarose-like G-LA units, with the latter exhibiting characteristic H1 anomeric proton resonance at 5.62 ppm [16]. The methylic protons in the cyclic pyruvate acetal bound in the C4 or C6 positions of G, assigned at 1.49/27.9 ppm, were determined at the highest concentration in fraction 1A, and their quantity decreased in 1B, 2B and 3A. Their presence was not confirmed in fraction 3B. Figure 1G-a shows the 2D HSQC NMR spectrum acquired for fraction GV-1B, highlighting the main assigned ^1^H/^13^C correlations. A 2D COSY NMR spectrum showing identified ^1^H/^1^H correlations is shown in Figure 1G-b. The comprehensive NMR analysis demonstrates that GV funorans represent structurally complex hybrid polysaccharides containing coexisting sulfated, methoxyl methoxylated, and acetylated domains. The relative abundance of these structural elements varies significantly depending on extraction method, fractionation protocol, and species origin.

### 2.2. Total Sugar, Sulfate, Protein, and Other Impurities

Comprehensive compositional analysis of purified GV fractions (GV-1A to GV-3B) revealed significant variations in biochemical constituents that correlate with extraction conditions (Table S1). Total sugar content, representing the core polysaccharide component, exhibited a substantial range from 50.18 ± 0.64% in GV-1A to 80.27 ± 7.69% in GV-1B, demonstrating the efficiency of the extraction protocol in recovering carbohydrate material. Notably, fractions GV-2B (78.61 ± 1.69%) and GV-3A (74.42 ± 0.92%) also contained elevated polysaccharide levels, while GV-1A showed comparatively reduced yield, suggesting temperature-dependent extraction efficiency. Sulfate content, a critical determinant of bioactivity in galactans, varied markedly between fractions (6.60 ± 0.05% to 21.59 ± 0.12%). The highest sulfation levels occurred in GV-1B (21.59 ± 0.12%), GV-2A (21.09 ± 0.08%%), and GV-3A (21.53 ± 0.31%), whereas GV-3B contained moderately lower sulfate content (16.66 ± 0.08%). These values confirm the successful isolation of sulfated galactans while highlighting that sulfate density does not necessarily correlate with extraction yield, as evidenced by the combination of low sugar but moderate sulfate content in GV-1A (6.60 ± 0.05%). This observation supports the emerging understanding that biological activity depends on complex structure–function relationships beyond simple sulfate quantification [17].

Protein contamination remained minimal across all fractions (0.47 ± 0.06% to 2.03 ± 0.12%), with GV-2B (2.03 ± 0.12%) and GV-1B (1.91 ± 0.21%) containing slightly elevated levels, potentially indicating residual glycoprotein complexes. While these trace proteins could influence bioactivity through synergistic effects, their low abundance suggests the observed biological properties primarily derive from polysaccharide constituents [18]. Total phenolic content (TPC) analysis revealed undetectable phenolic compounds in three fractions (GV-1A, GV-2A, GV-3A: ND) and only trace amounts in others (GV-1B: 0.07 ± 0.02%; GV-2B: 0.34 ± 0.03%; GV-3B: 0.17 ± 0.01%) (Figure 1E). These negligible values confirm effective removal of phenolic contaminants during purification, implying that any antioxidant activity stems from intrinsic polysaccharide features rather than co-extracted phenolics [19,20].

### 2.3. Macrophage Functional Responses

#### 2.3.1. MTT Assay

**Figure 2:**
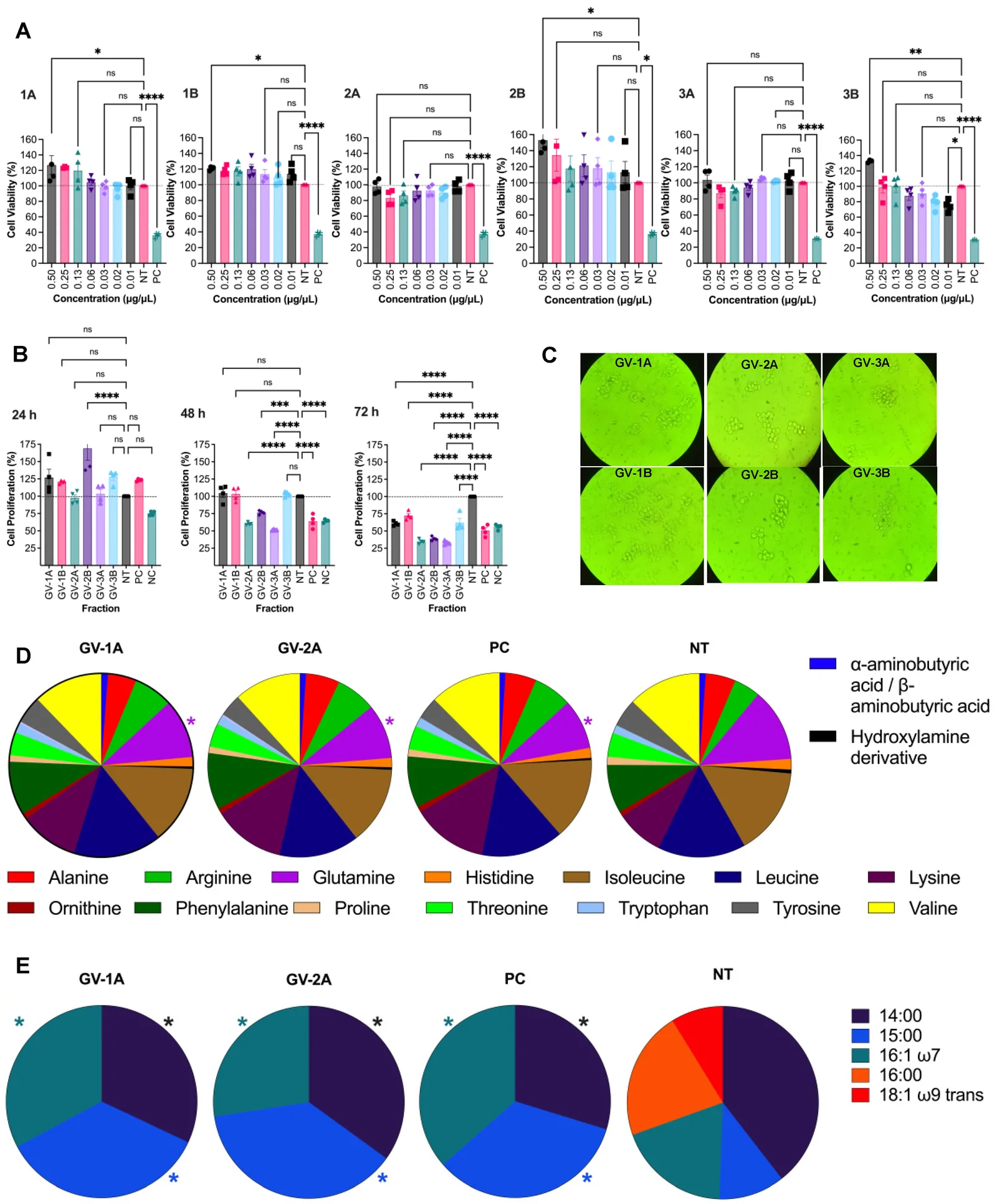
Bioactivity of *G. variabilis* (GV) funoran extracts in RAW264.7 macrophages. (**A**). Cytotoxicity after 24 h treatment with GV fractions: cold-extracted fractions 1A and 1B, and hot-extracted fractions 2A, 2B, 3A, and 3B (0.01–0.5 μg/μL) assessed by MTT assay. (**B**). Time-dependent proliferation after 24, 48, and 72 h treatment (0.5 μg/μL) assessed by AlamarBlue assay. (**C**). Representative bright-field micrographs (40×) of RAW264.7 macrophages after 72 h treatment illustrating changes in cell morphology and density. (**D**). Intracellular amino acid profiles after 24 h treatment with GV-1A and GV-2A (0.02 μg/μL). NT = non-treated control (Milli-Q water). Data represent relative abundance as percentage (%) of the total measured intracellular amino acid pool. (**E**). Fatty acid composition profiling after 24 h treatment with GV-1A and GV-2A (0.02 μg/μL). Pie charts show relative percentage composition of detected fatty acids: saturated (C14:0 myristate, C16:0 palmitate, C18:0 stearate), monounsaturated (C16:1 ω7 palmitoleate, C18:1 ω9 cis oleate, C18:1 ω9 trans elaidate), and polyunsaturated (C18:2 ω6 cis linoleate). All data are presented as mean ± SEM (*n* = 4). Statistical significance was determined by one-way ANOVA with Dunnett’s post hoc test versus NT: * *p* < 0.05, ** *p* < 0.01, *** *p* < 0.001, **** *p* < 0.0001.

In RAW264.7 macrophages, cold-extracted fractions GV-1A and 1B consistently enhanced cell proliferation across all concentrations (0.01–0.50 μg/μL), reaching 126.90 ± 12.20% and 120.70 ± 1.10% viability, respectively, at 0.50 μg/μL (Figure 2A). Our previous study also confirmed that the tested concentrations (0.01–0.50 μg/μL) are non-toxic and safe for cells, which confirms that the polysaccharide itself has higher molecular weight and lower toxicity effects [21]. Hot-extracted fractions exhibited more variable effects: GV-2A showed mild cytotoxicity at intermediate doses (83.30–86.50%), while GV-2B and GV-3B strongly promoted proliferation (153.00 ± 9.60% and 132.60 ± 1.0%, respectively, at 0.50 μg/μL). GV-3A demonstrated moderate enhancement with a viability dip to 86.97 ± 5.43% at 0.25 μg/μL. Based on efficacy and reproducibility, an optimal concentration of 0.50 μg/μL was found for GV-1B, GV-2B, GV-3A, and GV-3B, while 0.25 μg/μL is optimal for GV-1A. These results demonstrate that GV polysaccharide fractions exert fraction-specific effects on RAW264.7 macrophage viability. Cold-extracted fractions (GV-1A, GV-1B) consistently enhanced proliferation, while hot-extracted fractions showed divergent effects: GV-2B and GV-3B were strongly proliferative, whereas GV-2A and GV-3A exhibited mild cytotoxicity at higher concentrations. This fraction-dependent activity profile underscores the selective immunomodulatory properties of these marine polysaccharides in macrophages [22].

#### 2.3.2. Cell Proliferation

After 24 h of exposure, all fractions significantly increased cell proliferation compared to the untreated control (NT = 100%). The hot-extracted fraction GV-2B induced the most potent stimulation, achieving 169.0 ± 17.2% viability. Strong proliferative effects were also noted for the cold-extracted fractions GV-1A (126.9 ± 12.2%) and GV-1B (120.7 ± 1.1%), as well as the hot-extracted fraction GV-3B (127.9 ± 4.8%) (Figure 2B). These early proliferative responses align with reports on marine-derived polysaccharides, which often trigger initial macrophage activation via TLR4 or dectin-1 pathways [23]. By 48 h, a clear divergence emerged. Fractions GV-1A (104.6 ± 5.9%), GV-1B (103.3 ± 5.2%), and GV-3B (103.3 ± 1.8%) maintained viability levels comparable to NT. Conversely, fractions GV-2A (60.3 ± 0.9%), GV-2B (76.6 ± 1.4%), and GV-3A (51.6 ± 0.4%) exhibited substantial cytotoxicity. This cytotoxic effect was further exacerbated after 72 h, with GV-2A, GV-2B, and GV-3A reducing viability to 34.7 ± 1.6%, 38.9 ± 1.5%, and 33.2 ± 1.3%, respectively. Such delayed cytotoxicity has been observed in other polysaccharide studies, where prolonged exposure to certain molecular weight fractions or impurity profiles co-extracted phenolics or proteins, etc.) induces oxidative stress or mitochondrial dysfunction in macrophages [24]. Among all fractions, GV-1B showed the most favorable long-term profile in macrophages, retaining the highest viability at 72.5 ± 3.0% at the 72 h timepoint. This highlights the importance of time-course viability assessments for immunomodulatory polysaccharides, since early proliferative effects do not ensure long-term safety [25].

#### 2.3.3. Phagocytosis

Cold-extracted fractions GV-1A (138.4 ± 2.8%) and GV-1B (137.9 ± 5.1%) increased phagocytosis compared to the non-treated control (NT = 100%). The most potent effect was observed with the hot-extracted fraction GV-2B (149.4 ± 1.1%), which exceeded the positive control (PC: 115.5 ± 1.7%) and represented an approximately 50% enhancement over baseline. Fraction GV-3B also significantly stimulated phagocytosis (143.8 ± 4.6%) (Table S2). In contrast, fractions GV-2A (132.9 ± 3.9%) and GV-3A (132.3 ± 2.3%) elicited only moderate, non-significant increases. An inverse correlation was noted between phagocytosis and cell proliferation. Previously, a study by Premarathna et al. 2024 [7] indicated that certain seaweed-derived polysaccharides, such as carrageenan, may inhibit cell proliferation by increasing nitric oxide (NO) production.

Fractions GV-1A and GV-1B potently stimulated phagocytic activity but did not concurrently promote cell proliferation, indicating that the observed immune activation is independent of mitogenic effects. Furthermore, the robust phagocytic response induced by GV-2B was paralleled by its induction of nitric oxide (NO) production (4.59 ± 0.18 μM), confirming a state of classical macrophage activation. The differential potency among fractions, with GV-2B and GV-3B being highly active, while GV-2A and GV-3A were less so, suggests that specific structural features of the polysaccharides dictate their immunostimulatory capacity. Thus, our study has identified GV polysaccharide fractions (GV-1A, GV-1B, GV-2B, and GV-3B) which are potent stimulators of innate immune function in macrophages.

#### 2.3.4. NO Production

NO is a key inflammatory mediator produced by activated macrophages and plays an important role in innate immune responses [26,27]. Treatment with several GV fractions resulted in a highly significant increase in NO production compared to the control (NT) (Table S2). Specifically, GV-1B (37.03 ± 0.84 μM, *p* < 0.0001), GV-2B (33.86 ± 4.15 μM, *p* < 0.0001), and GV-3B (35.25 ± 2.94 μM, *p* < 0.0001) were the most potent inducers, generating NO levels significantly exceeding those induced by NT (3.32 ± 0.36 μM). The fraction GV-2A (27.28 ± 3.40 μM, *p* = 0.0002) also demonstrated a strong and significant effect. In contrast, the NO production induced by GV-1A (17.88 ± 0.42 μM, *p* = 0.0638) and GV-3A (18.24 ± 4.50 μM, *p* = 0.0524) was not statistically different from the control. As expected, GV-1B, GV-2A, GV-2B, and GV-3B induced NO levels significantly above the non-treated control group (NT = 3.32 ± 0.36 μM), whereas GV-1A and GV-3A did not show statistically significant NO induction. The ability of these GV fractions to promote a robust NO response highlights their potential as immunostimulatory agents that may contribute to enhancing innate immune defense [28].

The differential NO production among GV fractions is noteworthy. Fractions that induced high NO (GV-1B, GV-2B, GV-3B) were also strong inducers of phagocytosis, suggesting that NO production and phagocytic activity are functionally linked. However, the absence of NO induction by GV-1A did not impair stimulation of phagocytic activity, indicating that GV-1A promotes immune function through NO-independent mechanisms. This distinction is clinically relevant, as excessive NO production can contribute to tissue damage in chronic inflammation [26]. The ability of GV-1A to enhance phagocytosis without elevating NO may offer a safer immunomodulatory profile, avoiding the potential for collateral tissue injury associated with excessive NO. This observation aligns with our metabolomics data (Section 2.4), where arginine accumulation suggests suppression of iNOS activity.

### 2.4. Amino Acid Profile in RAW264.7 Macrophages: Induction of an Anti-Inflammatory Phenotype

The most pronounced and interpretable metabolic shift was observed in RAW264.7 macrophages, with compelling evidence indicating polarization toward an anti-inflammatory phenotype. In macrophages, arginine metabolism serves as a defining metabolic switch governing immune polarization [29]. Pro-inflammatory M1 macrophages utilize iNOS to convert arginine into NO, whereas anti-inflammatory M2 macrophages employ arginase to metabolize arginine into ornithine and polyamines [30].

Following polysaccharide treatment, we showed significant accumulation of intracellular arginine (NT: 4.61%, GV-1A: 6.87%, GV-2A: 7.06%) concurrent with a reduction in ornithine (NT: 0.88%, GV-1A: 1.04%, GV-2A: 0.96%) (Figure 2D). This accumulation strongly suggests that polysaccharide treatment inhibits arginase activity. While this metabolic signature diverges from canonical M2 polarization, it is a powerful indicator of an alternative anti-inflammatory mechanism [31]. By shunting arginine away from the arginase pathway, the polysaccharides may increase substrate availability for other immunoregulatory pathways or suppress M2-related proliferative processes. Critically, the pro-inflammatory iNOS/NO pathway was not activated. The concurrent modulation of both arginine utilization pathways results in a net anti-inflammatory effect.

Further supporting this finding, tryptophan levels remained stable across treatments (NT: 1.45%, GV-1A: 1.74%). As tryptophan catabolism is a hallmark of M1 activation, its stability further corroborates the absence of a robust pro-inflammatory response [32]. Lysine levels were higher in GV-2A-treated cells (12.70%) compared to NT controls (8.43%). Given the role of lysine in modulating inflammation and its requirement for protein synthesis during tissue repair, this elevation is consistent with an anti-inflammatory and pro-resolutive phenotype. The most significant evidence is arginine accumulation, indicative of arginase pathway suppression [30]. This points to a novel mechanism that bypasses classical M1/M2 polarization, instead establishing a unique metabolic state with a definitive net anti-inflammatory outcome.

The arginine accumulation phenotype observed here is distinct from canonical M2 polarization, where arginase converts arginine to ornithine and polyamines. This suggests that GV polysaccharides induce a unique metabolic state, neither classical M1 nor M2, that we term a ‘resolution-phase metabolic phenotype. This state is characterized by arginine sparing, which prevents NO overproduction while preserving arginine for other functions such as protein synthesis and polyamine production. Similar metabolic rewiring has been reported for other immunomodulatory agents, including specialized pro-resolving mediators (SPMs) and certain plant polysaccharides [33]. Our findings extend this concept to algal polysaccharides and suggest that GV fractions may offer a novel approach to promoting inflammation resolution without immunosuppression.

### 2.5. Fatty Acid Profile in RAW264.7 Macrophages: A Distinct Anti-Inflammatory Signature

Cellular fatty acid profiles offer critical insights into metabolic state and functional responses to bioactive compounds. We performed GC-MS analysis of fatty acid methyl esters (FAMEs) on RAW264.7 macrophages following 24 h treatment with GV-1A and GV-2A polysaccharides (Figure 2E). The results reveal distinct, fraction-specific alterations in lipid metabolism that correlate directly with the observed anti-inflammatory activity.

The most pronounced and interpretable shifts occurred in RAW264.7 macrophages, offering metabolic evidence consistent with the potent anti-inflammatory phenotype induced by GV polysaccharides (Table 2). NT control RAW264.7 cells were uniquely characterized by very high levels of methyl myristate (14:0, 35.52%) and methyl pentadecanoate (15:0, 10.01%). These fatty acids were completely absent in both GV-1A and GV-2A-treated cells. Treated cells showed a marked increase in palmitoleate (16:1 ω7) and palmitate (16:0) compared to NT. Elaidate (trans-9) levels were also higher in GV-treated RAW264.7 cells (GV-1A: 23.01%; GV-2A: 20.94%) compared to NT controls (7.94%).

This lipidomic profile is consistent with the anti-inflammatory and pro-resolution changes observed in whole-blood gene expression (*in vivo* qPCR data showing upregulation of Arg1, IL-10, TGF-β1). The complete disappearance of myristate (14:0) is highly significant, as myristic acid is a known potent activator of Toll-like receptor 4 (TLR4)/NF-κB signaling, a key driver of classical M1 pro-inflammatory activation [36,37]. Its removal from the cellular lipid pool strongly suggests fundamental suppression of this pathway. An increase in longer-chain fatty acids may indicate a shift toward oxidative phosphorylation and membrane remodeling, metabolic features commonly associated with M2-like, anti-inflammatory macrophages [38]. This shift away from a pro-inflammatory lipid milieu directly explains the potent *in vivo* efficacy of GV-1A and GV-2A, where they outperformed meloxicam by not merely inhibiting edema but actively reprogramming macrophage metabolism to resolve inflammation.

The elimination of myristate (14:0) from macrophage lipid pools is a particularly significant finding. Myristate is not only a TLR4 agonist but also a precursor for myristoylation, a post-translational modification that anchors proteins to membranes and regulates signaling [39]. Its complete absence suggests global remodeling of membrane protein localization and signaling pathways. This may explain the multi-faceted effects of GV fractions, including enhanced phagocytosis, altered cytokine production, and reduced inflammatory tone. Future studies should investigate whether GV fractions directly inhibit myristate synthesis or enhance its metabolism, potentially through the upregulation of fatty acid oxidation. The clinical relevance is underscored by the association between elevated circulating myristate and metabolic diseases, including type 2 diabetes and atherosclerosis [40]. GV fractions could therefore have applications beyond acute inflammation, extending to attenuation of metabolic syndrome and cardiovascular disease.

### 2.6. In Vivo Anti-Inflammatory Efficacy

The *in vitro* findings demonstrate that GV polysaccharides directly reprogram macrophage metabolism toward an anti-inflammatory phenotype. To determine whether these effects translate to therapeutic efficacy in a physiologically relevant context, we evaluated GV fractions in a carrageenan-induced paw edema model.

#### 2.6.1. Anti-Inflammatory Response to GV Extracts

**Figure 3:**
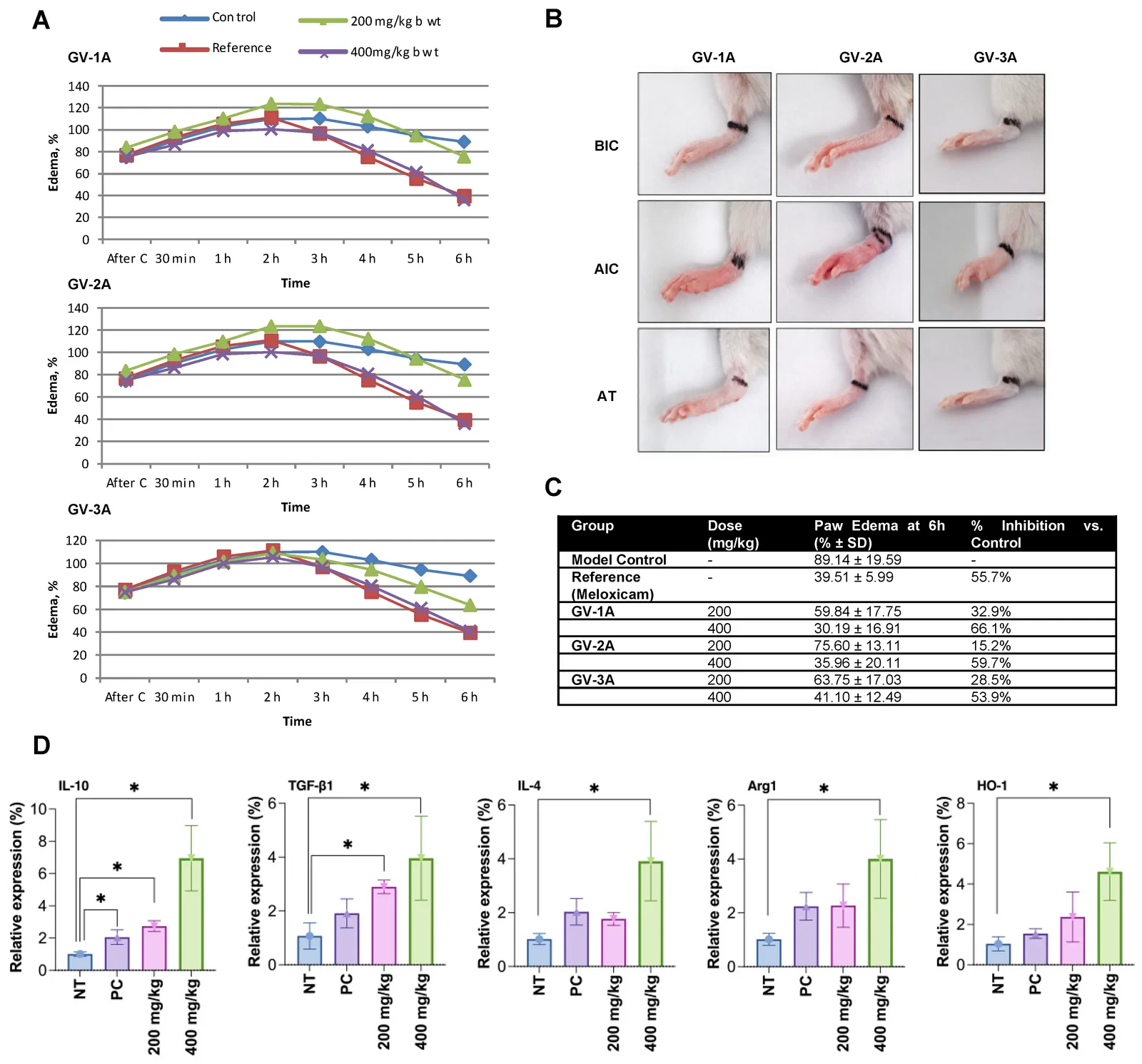
Effect of *G. variabilis* extracts on induced paw edema. (**A**). Time-course profile of mean paw edema (%) from 1 to 6 h after GV administration by gavage. The line graph illustrates the progression of inflammation and the differential effects of treatments over time. (**B**). The following chart visualizes the mean paw edema for all groups at the critical 6 h time point, providing a clear comparison of their efficacy. Cold fractions (GV-1A, GV-1B) were extracted at 25 °C; hot fractions (GV-2A, GV-2B, GV-3A, GV-3B) were extracted at 95 °C. (**C**). Anti-inflammatory efficacy at 6 h. (**D**). Relative cytokine expression using polysaccharide fractions: GV-2A (normalized using housekeeping gene; Glyceraldehyde-3-phosphate dehydrogenase (GAPDH), control; without treatment sample relative gene expression = 1). Data are presented as mean ± SEM (*n* = 4). One-way ANOVA determined statistical significance with Dunnett’s post hoc test comparing each fraction to the untreated control (NT, Milli-Q water) at each time point. * *p* < 0.05 versus NT.

The anti-inflammatory activity of three sulfated polysaccharide (SP) fractions from GV-1A (cold-water extract), GV-2A, and GV-3A (hot-water extracts) was evaluated in a carrageenan-induced paw edema model in mice (Figure 3A,B). The results, expressed as mean percentage increase in paw volume (±standard deviation), demonstrate a clear time-dependent and dose-dependent response for all fractions compared to the model control group (Figure 3C).

#### 2.6.2. Time Effect of Inflammation

In the control group, paw edema increased sharply, peaking around 2–4 h after carrageenan injection (110.06%) and then slowly subsiding but remaining high (89.14%) at 6 h, confirming a robust inflammatory response. This pattern is characteristic of carrageenan-induced acute inflammation, where the early phase (0–2 h) is mediated by histamine and serotonin, and the later phase (2–6 h) is driven by prostaglandins, bradykinin, and leukotrienes [41]. For every fraction administered by gavage (GV-1A, GV-2A, GV-3A), the 400 mg/kg dose resulted in significantly lower paw edema at the 6 h mark compared to its corresponding 200 mg/kg dose. This clear dose–response relationship strongly indicates that the observed anti-inflammatory effect is directly attributable to the administered fractions, consistent with previous reports on bioactive polysaccharides from marine organisms [42]. At the 400 mg/kg dose, all fractions exhibited significant anti-inflammatory activity. GV-1A was the most potent, showing the strongest inhibition (66.1%, edema: 30.19%). GV-2A was the second-most potent (59.7% inhibition, edema: 35.96%). GV-3A was the least potent of the three, though still effective (53.9% inhibition, edema: 41.10%). The efficacy of GV-1A at 400 mg/kg (66.1% inhibition) surpassed that of the standard NSAID drug meloxicam (55.7% inhibition), highlighting its exceptional therapeutic potential. This suggests that GV-1A may act through multiple synergistic pathways, possibly involving COX-2 suppression, NF-κB modulation, or cytokine regulation [43]. However, differences in oral bioavailability and absorption kinetics between the small-molecule NSAID meloxicam and high-molecular-weight GV polysaccharides may partly contribute to the observed differences in edema time-course.

#### 2.6.3. qPCR Analysis of Anti-Inflammatory Gene Expression

qPCR analysis of blood samples revealed that all three test compounds (GV-1A, GV-2A, and GV-3A) significantly modulated the expression of anti-inflammatory genes in a dose-dependent manner following carrageenan-induced paw edema. Treatment with GV-1A resulted in robust upregulation of all five anti-inflammatory markers. The high dose (400 mg/kg) group exhibited the most pronounced effects: *IL-10* (~15-fold), *TGF-β1* (~9.8-fold), *IL-4* (~11.3-fold), *Arg1* (~5.7-fold), and HO-1 (~7.0-fold) relative to the model control. Similarly, GV-2A treatment (400 mg/kg) showed substantial upregulation: *Arg1* (~15-fold), *IL-10* (~7-fold), *HO-1* (~6.9-fold), *IL-4* (~8-fold), and *TGF-β1* (~10-fold). GV-3A also produced an anti-inflammatory response, upregulating *IL-4* (~4.6-fold), *TGF-β1* (~3.0-fold), and *HO-1* (~3.0-fold), with more modest increases in *IL-10* (~1.5-fold) and *Arg1* (~2.7-fold). The reference drug meloxicam induced only moderate upregulation of these markers.

These data provide consistent molecular evidence that GV-derived compounds, particularly GV-1A and GV-2A, exert potent anti-inflammatory effects by actively promoting a pro-resolution immunological phenotype. The marked upregulation of *IL-10*, *IL-4*, and *TGF-β1* suggests a fundamental immunomodulatory mechanism. Strong *IL-10* induction indicates robust activation of immunoregulatory pathways that attenuate pro-inflammatory cytokine production and facilitate resolution [44,45]. The increase in *IL-4* and *TGF-β1* highlights a coordinated shift toward a Th2-dominated, anti-inflammatory environment conducive to tissue repair [46,47].

Crucially, substantial upregulation of Arginase-1 (*Arg1*), a canonical M2 macrophage marker, demonstrates that these compounds influence innate immune cell programming. The M1-to-M2 transition is pivotal for inflammation resolution and tissue remodeling [48,49]. This effect is complemented by induction of heme oxygenase-1 (*HO-1*), known for its cytoprotective and antioxidative roles during inflammatory stress [50,51]. The combined upregulation of *Arg1* and HO-1 underscores a dual-action mechanism that suppresses inflammatory damage while enhancing cellular resilience.

The differential efficacy among compounds is noteworthy. GV-1A and GV-2A exhibited markedly greater potency than GV-3A. Importantly, at 400 mg/kg, all three compounds outperformed meloxicam, which showed only moderate upregulation of resolution-associated genes. While meloxicam primarily acts as a COX inhibitor, *G. variabilis* compounds appear to suppress edema through multiple converging mechanisms: elimination of the TLR4 agonist myristate, lack of iNOS-driven NO production, and HO-1-mediated protection of endothelial barrier integrity. Concurrently, they actively upregulate resolution-phase markers *(Arg1*, *IL-10*, *IL-4*, *TGF-β1*), reflecting a transition from symptomatic anti-inflammation to true inflammation resolution [52,53].

### 2.7. Structure–Activity Relationships and Proposed Mechanism

The bioactivity of *G. variabilis* polysaccharides is fraction-specific and dictated by extraction-associated structural properties. Cold-water fraction GV-1A (~360 kDa, 6.6% sulfate) enhanced macrophage immune function *in vitro* and exhibited exceptional anti-inflammatory efficacy *in vivo*, rivaling meloxicam. Its potency is attributed to low molecular weight (binding to specific receptors, etc.) and unique pyruvate acetal content [3,54,55]. Hot-water fraction GV-2A (1480 kDa, 21.1% sulfate) functioned as a potent immunostimulant in macrophages, likely via Toll-like receptor engagement [56], although direct receptor binding studies are needed to confirm this. The inverse relationship between GV-1A (low sugar, moderate sulfate) and GV-2A (high sugar, high sulfate) illustrates how extraction conditions enable selective recovery of fractions with different properties. All fractions showed excellent safety in macrophages.

Mechanistically, GV treatment drives a pronounced immunometabolic shift in RAW264.7 macrophages: arginine accumulation (4.61% to 7.06%) with reduced ornithine, suggesting functional arginase inhibition, and complete elimination of pro-inflammatory myristate (14:0) and pentadecanoate (15:0), which is consistent with the suppression of TLR4/NF-κB signaling and promotion of pro-resolution polarization [57]. However, direct receptor binding studies (surface plasmon resonance, competitive binding assays, etc.) and downstream signaling analyses (Western blot for NF-κB phosphorylation, etc.) are required to definitively establish these mechanisms.

**Figure 4:**
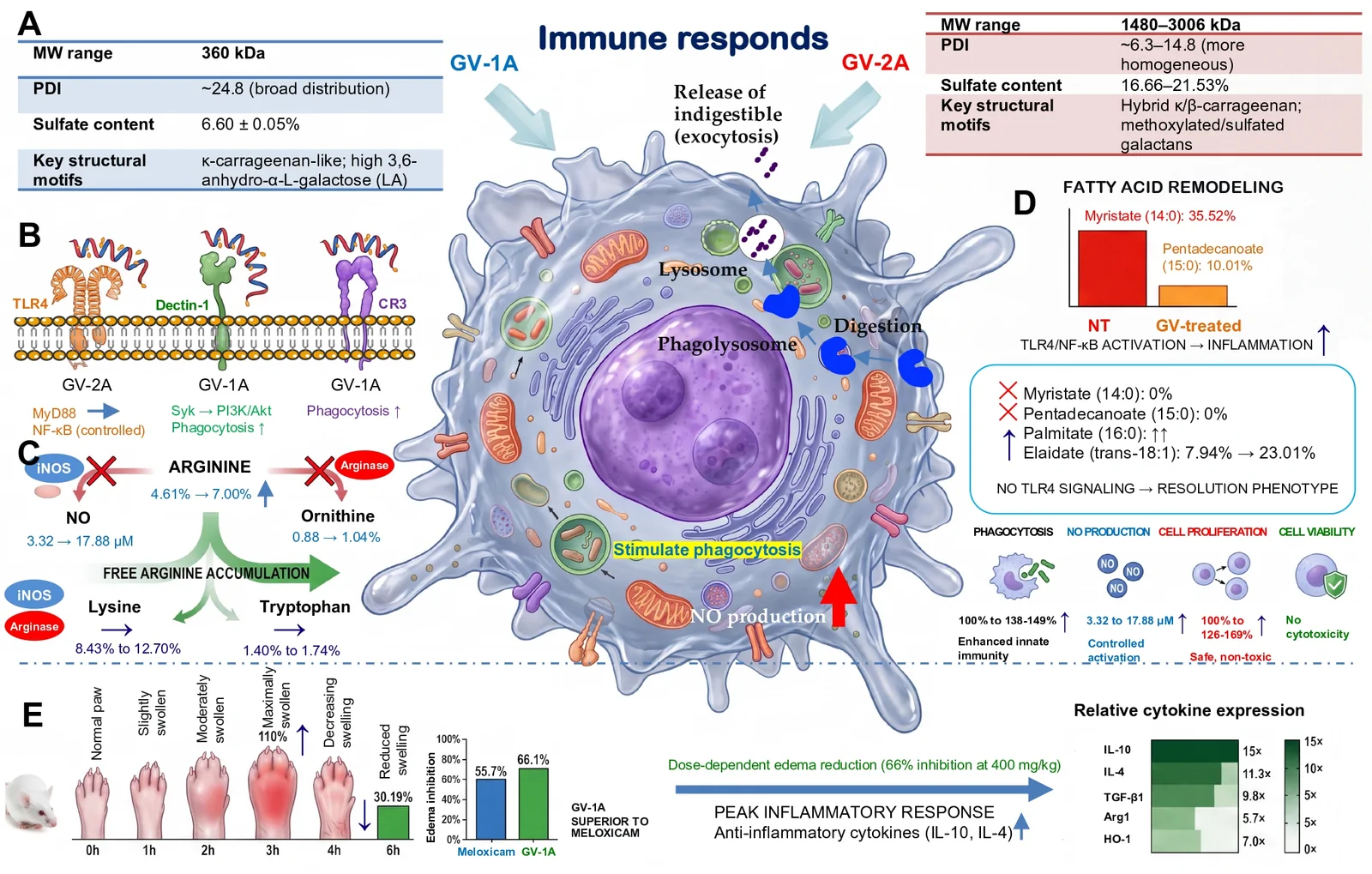
Mechanistic model for GV polysaccharide-mediated inflammation resolution. (**A**). Structural features of cold-extracted (GV-1A) and hot-extracted (GV-2A) polysaccharides from *G. variabilis*, showing differences in molecular weight, sulfation, etc. (**B**). Proposed receptor engagement (hypothetical model): low-MW GV-1A engages Dectin-1/CR3 (consistent with enhanced phagocytosis), while high-sulfate GV-2A may engages TLR4 (consistent with immunostimulatory effects). These receptor assignments are inferred from structural features and functional responses, but direct receptor binding studies have not been performed. Both pathways are hypothesized to lead to downstream signaling and metabolic reprogramming. (**C**). Immunometabolic reprogramming in macrophages: GV treatment induces arginine accumulation (4.61% to 7.06%) via iNOS/arginase suppression, (**D**). eliminates pro-inflammatory myristate (35.52% to 0%), and increases palmitate/elaidate, shifting macrophages toward a resolution phenotype. (**E**). *In vivo* validation: GV-1A (400 mg/kg) outperforms meloxicam in edema reduction (66.1% vs. 55.7%) and upregulates resolution markers (*IL-10*, *IL-4*, *TGF-β1*, *Arg1*, HO-1). Arrow symbols: ↑, increased; ↓, decreased.

This *in vitro* metabolic signature, distinct from canonical M2 polarization, is consistent with the hypothesis that GV polysaccharides suppress arginine consumption through the arginase pathway in macrophages (see Figure 4), potentially preventing excessive NO production while preserving arginine for other functions. *In vivo*, GV treatment was associated with transcriptional upregulation of *Arg1* (5-15-fold), alongside *IL-10*, *IL-4*, *TGF-β1*, and HO-1. We interpret this as a tissue-level anti-inflammatory response that may involve *Arg1* expression from multiple cell types, not exclusively macrophages.

These complementary findings, macrophage-autonomous arginine sparing *in vitro* versus systemic *Arg1* upregulation *in vivo*, support a working model whereby GV polysaccharides promote inflammation resolution through context-dependent mechanisms. We propose the term “resolution-phase metabolic phenotype” to describe this integrated response, recognizing that this is a hypothesis-generating framework rather than an established paradigm. This phenotype encompasses (1) macrophage metabolic reprogramming (arginine sparing, myristate elimination), (2) tissue-level anti-inflammatory gene expression (*IL-10*, *Arg1*, HO-1), and (3) functional resolution of inflammation (edema reduction). Future studies using tissue-specific Arg1 knockout models and metabolic flux analysis with stable isotope tracers will be required to definitively establish the causal relationships between these observations.

Biological activity arises from an integrated combination of molecular weight, sulfate content, and molecular architecture rather than any single parameter [58]. This study establishes *G. variabilis* sulfated polysaccharides, with GV-1A and GV-2A as lead compounds, as promising immunometabolic agents for inflammation resolution (Table 3).

Integration of these data reveals a multi-tiered mechanism wherein structural features of GV-1A, including low molecular weight (~360 kDa), specific sulfate/methoxyl patterns, and pyruvate acetal content, facilitate cellular uptake or membrane interaction. This initiates a cascade of metabolic reprogramming: arginase/iNOS suppression leads to arginine preservation, preventing excessive NO production, while myristate elimination removes a TLR4-driven pro-inflammatory signal. Consequently, macrophages exhibit enhanced phagocytic capacity without inflammatory overactivation, and *in vivo*, this translates to edema resolution through upregulation of *IL-10*, *TGF-β1*, *Arg1*, and HO-1. Particularly, unlike COX-inhibiting meloxicam, GV polysaccharides actively drive a pro-resolving transcriptional program, positioning them as agents that promote changes consistent with resolution, rather than conventional suppressors of inflammation.

## 3. Study Limitations and Future Directions

The precise molecular receptors initiating immunometabolic reprogramming remain unidentified, and direct receptor binding studies (SPR, competitive binding) are required. While the *in vivo* study achieved statistical significance with *n* = 4 per group, larger sample sizes would increase statistical accuracy. Future studies should use receptor blockade (anti-TLR4 antibodies, pharmacological antagonists) combined with stable isotope-labeled tracers (^13^C-arginine, ^13^C-glutamine) to establish causality, and extend efficacy evaluation to chronic inflammatory models (colitis, arthritis) to assess long-term therapeutic potential. Additionally, quantitative correlations between specific structural parameters, sulfate ester positioning, degree of sulfation, anhydrogalactose content, monosaccharide composition, and molecular conformation, and their corresponding pharmacological endpoints, warrant systematic investigation.

A critical limitation of this study is the use of different systems for *in vitro* metabolomics (RAW264.7 macrophages) and *in vivo* gene expression analysis (whole blood). Although both systems demonstrated anti-inflammatory outcomes, the apparent discrepancy in arginine metabolism, specifically arginine accumulation *in vitro* vs. *Arg1* upregulation *in vivo*, reflects the fundamentally different cellular compositions and microenvironments of these systems. RAW264.7 cells represent a homogeneous macrophage population with cell-autonomous responses, whereas whole blood contains multiple immune cell types whose integrated responses may include *Arg1* expression from non-macrophage sources. The metabolomics dataset was also collected at a single concentration and single timepoint (24 h) for only two representative fractions, providing a preliminary snapshot rather than a comprehensive characterization of metabolic dynamics.

To directly validate the mechanistic model proposed in this study, we have committed to the following specific future experiments: (1) primary macrophage validation using bone marrow-derived macrophages to confirm that the arginine accumulation and myristate elimination phenotypes are macrophage-autonomous; (2) tissue-specific metabolic analysis of paw tissue from treated animals to measure arginine and ornithine levels by LC-MS and *Arg1* enzymatic activity; (3) cell-type-specific Arg1 expression analysis using flow cytometry to identify the cellular sources of *Arg1* (macrophages, neutrophils, endothelial cells); (4) temporal profiling of metabolic reprogramming at multiple timepoints (12, 24, 48 h); and (5) metabolic flux analysis using ^13^C-arginine stable isotope tracers to directly measure arginine consumption and ornithine production. These experiments are essential to validate the working model presented in Figure 4 and will be reported in follow-up studies.

## 4. Materials and Methods

### 4.1. Seaweed Material and Preparation

*G. variabilis* (GV) specimens were collected from the coastal waters of Sri Lanka in 2021. Immediately after collection, the biomass underwent sequential washing with (1) seawater to remove surface debris, (2) fresh water to eliminate residual sand and epiphytic organisms, and (3) filtered tap water for final purification. The cleaned algal material was lyophilized (Model Scanvac, Brøndby, Denmark) to a constant dry weight and subsequently ground using a water-cooled milling system (IKA^®^ A10, Staufen, Germany) to prevent the thermal degradation of heat-sensitive compounds. The resulting powder was stored in vacuum-sealed bags at −20 °C until further use.

### 4.2. Funoran Extraction from Gelidiopsis Variabilis

**Figure 5:**
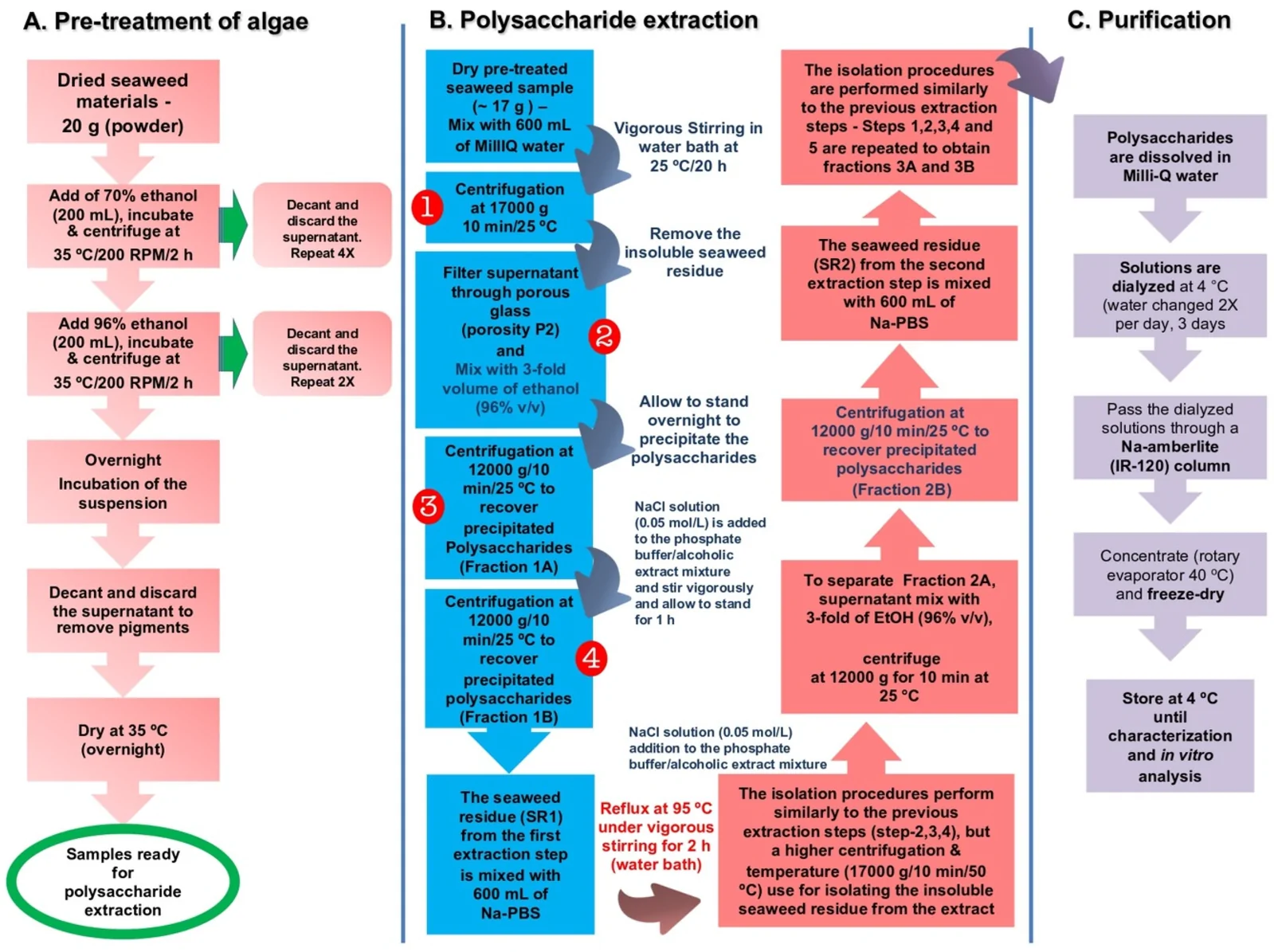
Schematic representation of the pre-treatment (**A**), extraction (**B**) and purification (**C**) procedures used to obtain the different polysaccharide fractions. SR: Seaweed Residue.

For the removal of low-molecular-weight contaminants (pigments, proteins, and lipids), 20 g of lyophilized GV powder was subjected to sequential ethanol extraction (70% *v*/*v*, 35 °C, 2 h, twice; then 96% *v*/*v*, 35 °C, overnight). Purified biomass was dried (35 °C) and stored at 4 °C. Polysaccharides were extracted from depigmented biomass (~17 g) using sequential cold (25 °C, 20 h) and hot (95 °C, 2 h) extraction in 20 mM sodium phosphate buffer (pH 9). Fractions were precipitated with 96% ethanol (3:1 *v*/*v*) without or with 50 mM NaCl supplementation (Figure 5). Crude polysaccharide fractions were purified by dialysis (14 kDa MWCO) against Milli-Q water for 72 h (water changed every 12 h, 1:100 *v/v* sample-to-water ratio), followed by cation-exchange chromatography (Amberlite IR-120, Na^+^ form) to convert polysaccharides to their sodium salts. Purified fractions were concentrated by rotary evaporation (40 °C), lyophilized, and stored in desiccated amber vials at 4 °C [19,21].

### 4.3. Characterization of Extracts

#### 4.3.1. Size-Exclusion Chromatography

Molecular weights were determined by HPLC-SEC (Shodex OHpak SB-806MHQ columns, 60 °C; 0.1 M NaNO_3_ mobile phase, 0.8 mL/min) (Shimadzu, Kyoto, Japan) calibrated with pullulan standards (0.342–2400 kDa) as described previously [19]. Mw, Mn, and polydispersity were calculated using Shimadzu LabSolutions GPC software version 5.97 (Shimadzu, Kyoto, Japan).

#### 4.3.2. Fourier Transform Infrared Spectrometer (FTIR) Analysis

FTIR spectra were acquired using a Nicolet iS50 spectrometer (Thermo Fisher Scientific, Waltham, MA, USA) (400–4000 cm^−1^, 4 cm^−1^ resolution, 64 scans) at 25 °C as previously described [19]. Data were processed using Omnic software (v.9.2).

#### 4.3.3. Nuclear Magnetic Resonance (NMR) Spectroscopy

NMR samples were prepared as previously described [19]. Briefly, GV polysaccharides (0.2% *w*/*v* in Milli-Q water) were ultrasonicated (Qsonica Q700, Newtown, CT, USA, 3 mm tip, 70 W, 80 kHz, 50% amplitude, 30 min), lyophilized, and redissolved in D_2_O (1.5% *w*/*v*) containing 10 mM DSS as internal reference. ^1^H and ^13^C NMR spectra were acquired on an Agilent DD2 spectrometer (Agilent Technologies, Santa Clara, CA, USA) (500 MHz for ^1^H, 125 MHz for ^13^C) at 65 °C. ^1^H spectra used a 90° pulse (2.5 s acquisition, 25 s relaxation delay, 64 scans); ^13^C spectra used 45,000 scans. 2D COSY and HSQC experiments were also performed. Spectra were processed with MestreNova (v14.3.2) and referenced to DSS (0 ppm).

### 4.4. Determination of Total Sugar, Sulfate, and Other Impurities (Protein)

The chemical composition of GV polysaccharide fractions was systematically characterized through a series of validated analytical methods. Total carbohydrate content was quantified using the phenol–sulfuric acid colorimetric assay according to established protocols [59], with absorbance measurements performed at 490 nm using a Shimadzu UV-1800 spectrophotometer (Shimadzu Corporation, Kyoto, Japan). Sulfate group content was determined through a modified barium chloride-gelatin method [60], where samples were hydrolyzed with 1 M HCl prior to precipitation with BaCl_2_. Total phenolic compounds were assessed using an optimized Folin–Ciocalteu procedure [61], with absorbance measured at 765 nm after 30 min incubation at room temperature. Protein contamination levels were evaluated using a bicinchoninic acid (BCA) assay kit (Ref. 23227, Thermo Scientific, Waltham, MA, USA) following the manufacturer’s protocol, with bovine serum albumin as the calibration standard.

### 4.5. In Vitro Study

#### 4.5.1. Cell Culture Maintenance

RAW264.7 macrophages (ECACC, 91062702, Salisbury, UK) were cultured in DMEM supplemented with 10% FBS, 1% penicillin–streptomycin, and 2 mM L-glutamine at 37 °C in 5% CO_2_, as described previously [21,62].

#### 4.5.2. MTT and AlamarBlue Assays

RAW264.7 macrophages were seeded in 96-well plates (2 × 10^4^ cells/well) and treated with GV fractions (0.01–0.5 μg/μL) for 24 h. Cell viability was assessed by MTT assay (0.5 mg/mL) as described previously ([21]. Absorbance was measured at 570 nm (reference 630 nm), and viability was normalized to untreated controls. For proliferation assessment, RAW264.7 macrophages (2 × 10^4^ cells/well) were treated with GV fractions (0.5 μg/μL) for 24–72 h. Cell proliferation was measured using the alamarBlue assay according to established protocols [21,63]. LPS (100 ng/mL) and mitomycin-C (0.04 μg/μL) served as positive and negative controls, respectively.

#### 4.5.3. Nitric Oxide Production and Phagocytosis

RAW264.7 macrophages (1 × 10^5^ cells/well) were treated with GV fractions (0.01 μg/μL) for 24 h. Nitrite accumulation in culture supernatants was measured using the Griess reagent, and phagocytic activity was assessed by neutral red uptake, as described previously [21,64]. LPS (10 μg/mL) served as a positive control. Absorbance was measured (FLUOstar OPTIMA microplate reader, BMG LABTECH, Wiesbaden, Germany) at 540 nm, and activity was expressed as a percentage of the untreated control.

### 4.6. Amino Acid and Fatty Acid Metabolomics in Polysaccharide-Treated Cell Lines

#### 4.6.1. Cell Sample Collection for Metabolomic Analysis

RAW264.7 cells were seeded at a density of 2 × 10^5^ cells/well in 6-well plates and maintained under standard conditions (37 °C, 5% CO_2_) for 24 h. The culture medium was then replaced with 500 µL of fresh DMEM containing GV polysaccharide extracts (GV1A or GV2A) at a final concentration of 0.02 µg/µL, or a control (Milli-Q water, NT). After a further 24 h incubation, the medium was aspirated, and the cells were washed gently with cold phosphate-buffered saline (PBS) to remove residual media and extracellular metabolites. Cell pellets were collected and stored at −80 °C until metabolite extraction. To avoid confounding metabolomic comparisons by treatment-induced differences in cell proliferation, samples were normalized to equal cell numbers prior to metabolite extraction. Following treatment, cells were detached by trypsinization, stained with 0.4% (*w*/*v*) trypan blue, and counted using a hemocytometer (Sigma-Aldrich, Munich, Germany). Equal numbers of viable cells (2 × 10^6^) from each treatment group were subsequently collected and pelleted for extraction.

#### 4.6.2. Amino Acid Extraction and Derivatization

Intracellular amino acids were extracted based on established methods with modifications [65]. Cell pellets were washed with PBS, centrifuged (1500× *g*, 5 min, 4 °C), and the supernatant was discarded. The pellet was resuspended in 250 µL of ultrapure water containing 5-methyl-DL-tryptophan as an internal standard to correct for procedural losses. Cells were lysed, and metabolites were extracted by sonication for 20 min. To precipitate proteins and separate phases, 250 µL of methanol and 500 µL of chloroform were added. After centrifugation (2000× *g*, 10 min), the upper aqueous phase containing hydrophilic metabolites was collected. This extract was evaporated to dryness under nitrogen gas at 50 °C for concentration, and the residue was reconstituted in 50 µL of ultrapure water. For LC-MS analysis, derivatization was performed as described by Oldekop et al. 2014 [66]. Briefly, a 50 µL aliquot was reacted with 75 µL of a DEEMM working solution (1:50 *v*/*v* in methanol) and 175 µL of borate buffer (pH 9.0). The mixture was incubated in the dark at room temperature overnight to ensure complete derivatization.

#### 4.6.3. Liquid Chromatography–Mass Spectrometry (LC-MS) Analysis

DEEMM-derivatized amino acids (prepared as described in Section 4.6.2) were analyzed using an Agilent 6230 Q-TOF mass spectrometer (USA). Chromatographic separation was performed using an Agilent Poroshell 120-EC-C-18 column (3.0 mm × 50 mm, 2.7 µm) at 25 °C. The mobile phase was (A) water/acetonitrile (95:5 *v*/*v*) with 0.1% formic acid and 10 mM ammonium acetate and (B) acetonitrile/water (95:5) with 0.1% formic acid. The flow rate was set at 0.5 mL/min using a gradient elution system with solvent mixture A. The gradient program was as follows: 0–5 min, 90% A to 73% A; 5–12 min, 73% A; 12–14 min, 73% A to 0% A; 14–17 min, 0% A; and 17–20 min, 0% A to 90% A. The injection volume was 5 µL. Mass spectrometry was performed with electrospray ionization in positive mode. Source parameters were dry gas, 10 L/min at 325 °C; nebulizer, 480 kPa; capillary voltage, 4000 V. The intracellular levels of five target amino acids (α-alanine, β-alanine, isoleucine, leucine, and tryptophan) were quantified using external calibration curves from commercially available standards (Sigma-Aldrich, St. Louis, MO, USA). In addition, an amino acid standard mixture (AAS18, Sigma-Aldrich, USA) was used to validate peak identification and ensure accurate quantification of all detected amino acids.

#### 4.6.4. Gas Chromatography–Mass Spectrometry (GC-MS) Analysis

The fatty acid profile of RAW264.7 cells treated with polysaccharides (GV-1A and GV-2A, etc.) was analyzed via gas chromatography–mass spectrometry (GC-MS) [67]. Briefly, frozen cell pellets were stored at −80 °C until analysis. Lipids were extracted using a chloroform:methanol (2:1, *v*/*v*) mixture. Each cell sample was homogenized with 250 µL of methanol and 500 µL of chloroform, followed by vigorous vortexing for 90 s and a 30 min incubation in an ultrasonic bath at 40 °C. After adding 200 µL of a 2% NaCl solution, the mixture was vortexed and centrifuged at 1000× *g* for 10 min. The lower chloroform layer was collected and dried under a stream of nitrogen at 35 °C. Fatty acid methyl esters (FAMEs) were then prepared by adding 150 µL of a 5% sulfuric acid solution in methanol to the dried extracts. The mixture was derivatized at 50 °C for 1 h (in a water bath), with gentle shaking every 15 min. After cooling, the FAMEs were extracted by adding 100 µL of water and 150 µL of hexane, followed by vortexing and phase separation. The hexane layer was transferred to a new vial, dried under nitrogen, and reconstituted in 100 µL of hexane for GC-MS analysis.

FAME quantification was performed on a Shimadzu GCMS-QP2010 Ultra system (Kyoto, Japan) equipped with a Phenomenex ZB-5MS capillary column (30 m × 0.25 mm, 0.25 µm). Helium was used as the carrier gas at a flow rate of 30 cm/s. The injection volume was 1 µL in split mode (split ratio 1:100). The oven temperature program was as follows: initial temperature 160 °C, increased to 260 °C at 2.5 °C/min, then to 298 °C at 5 °C/min, and held for 15 min. Fatty acids were identified by comparing their retention times and mass spectra to commercial standards (37 FAME Mix from Supelco CRM47885, Sigma-aldrich, Germany). Results are expressed as the mass percentage of each fatty acid relative to the total identified.

### 4.7. In Vivo Assay

#### 4.7.1. Experimental Animals and Ethical Statement

Healthy adult male Swiss albino mice weighing 25.5 ± 2.5 g were used for the study. The animals were housed in standard polypropylene cages under controlled environmental conditions, including a temperature range of 21–26 °C, relative humidity of 60–67%, a ~12 h light/dark cycle, and approximately fresh air changes per hour. They were provided with a standard rodent diet and water ad libitum. Prior to the experiment, all animals were acclimatized to the laboratory conditions for one week.

All experimental procedures were approved by the Institutional Animal Ethics Committee (IAEC) on 17 February 2024 (Approval No. MB/IAEC/2024/02/17B) and conducted at Mass Biotec Pvt. Ltd. in strict compliance with the guidelines of the Organization for Economic Co-operation and Development (OECD).

#### 4.7.2. Preliminary Toxicity Study

A preliminary dose–response study was conducted to determine the acute toxic effects of the GV algal fractions (polysaccharide) on mice. Graded doses of each fraction (200 mg/kg, and 400 mg/kg body weight) were orally administered to mice once daily for 14 days. Animals were observed individually for the first 45 min, periodically for the first 24 h, and then daily for 14 days for any signs of morbidity, mortality, or behavioral changes (including lethargy, piloerection, or altered locomotion). The results indicated that all tested dosages of the algal fractions were well-tolerated and not toxic to the mice. Therefore, the doses of 200 mg/kg and 400 mg/kg were selected for the anti-inflammatory study.

#### 4.7.3. Study Design for Anti-Inflammatory Activity

The anti-inflammatory activity of GV fractions (GV-1A, GV-2A, GV-3A) was evaluated using a carrageenan-induced paw edema model. Mice were randomly divided into eight groups (*n* = 4 per group). Group I (negative control) received vehicle (1% carboxymethyl cellulose, CMC) orally. Group II (positive control) received the standard anti-inflammatory drug meloxicam orally (10 mg/kg). Treatment groups III–VIII received GV fractions orally at 200 or 400 mg/kg body weight as follows: Group III, GV-1A (200 mg/kg); Group IV, GV-1A (400 mg/kg); Group V, GV-2A (200 mg/kg); Group VI, GV-2A (400 mg/kg); Group VII, GV-3A (200 mg/kg); Group VIII, GV-3A (400 mg/kg). One hour after oral administration, acute inflammation was induced by subcutaneous injection of 50 µL of 1% (*w*/*v*) carrageenan into the plantar surface of the right hind paw (detailed in Section 4.7.4). Paw volumes were measured at 0.5, 1, 2, 3, 4, 5, and 6 h post-induction.

#### 4.7.4. Induction of Inflammation and Measurement of Paw Edema

After random allocation into experimental groups, mice received their respective treatments orally via gavage. One hour post-treatment, acute inflammation was induced by a subcutaneous injection of 50 µL of 1% (*w*/*v*) carrageenan solution in sterile saline into the plantar surface of the right hind paw [68,69]. The paw volume was measured using a digital plethysmometer immediately before carrageenan injection (baseline volume, V_0_) and subsequently at 0.5, 1, 2, 3, 4, 5, and 6 h after the injection. For consistency in measurement, the right hind paw was marked approximately 2 cm from the tip. The degree of paw edema was determined as the increase in paw volume relative to the baseline value (ΔV = Vt − V_0_).

% Inhibition = [(Vc − Vt)/Vc] × 100

where Vc is the mean edema volume in the model control group, and Vt is the mean edema volume in the treated group.

#### 4.7.5. Isolation of RNA, Preparation of cDNA, and qPCR to Detect Cytokine Gene Expression

##### RNA Isolation from Blood Samples

To investigate the molecular mechanisms of the test compounds (GV-1A, GV-2A, GV-3A), the expression of key anti-inflammatory genes was quantified in blood from the paw edema model. At 6 h post-carrageenan induction, which corresponds to the peak of the inflammatory response, approximately 20–50 μL of blood was collected from the tail vein using sterile 25G lancets and transferred into RNase-free EDTA tubes. Total RNA was extracted using the Qiagen RNeasy Mini kit (North Rhine-Westphalia, Germany), incorporating an on-column DNase I digestion step to eliminate genomic DNA contamination, as per the manufacturer’s protocol.

##### cDNA Synthesis and Quantitative PCR (qPCR)

Complementary DNA (cDNA) was synthesized from 500 ng of total RNA using the SuperScript IV First-Strand Synthesis Kit (Thermo Fisher Scientific) with a combination of random hexamers and oligo(dT) primers in a 20 μL reaction volume. No-reverse transcription controls were included to confirm the absence of genomic DNA amplification. Quantitative real-time PCR was performed using SYBR Green Master Mix (Applied Biosystems) on a QuantStudio™ 5 Real-Time PCR System (Thermo Fisher). Each 20 μL reaction consisted of 10 μL of 2× SYBR Green Master Mix, 0.3 μM of each gene-specific primer, and 2 μL of cDNA template. All samples were run in technical triplicate. The thermocycling protocol was as follows: initial denaturation at 95 °C for 3 min; 40 cycles of 95 °C for 10 s and 60 °C for 30 s (data acquisition phase); followed by a melt curve analysis from 65 °C to 95 °C with an increment of 0.5 °C every 5 s. The following target genes were analyzed: Interleukin-10 (*IL-10*), Transforming Growth Factor-beta 1 (*TGF-β1*), Interleukin-4 (*IL-4*), Arginase-1 (*Arg1*), and Heme Oxygenase-1 (HO-1). Glyceraldehyde-3-phosphate dehydrogenase (*GAPDH*) was used as a housekeeping gene for normalization. Relative gene expression was calculated using the 2(−ΔΔCt) method [70,71], where the fold change was normalized to GAPDH and expressed relative to the vehicle-treated control (model) group.

### 4.8. Statistical Analysis

Statistical analyses were performed using GraphPad Prism software (version 10.0.2, GraphPad Software Inc., San Diego, CA) on macOS. Experimental data from cellular assays were expressed as mean ± standard error of the mean (SEM) derived from four independent biological replicates. One-way ANOVA was used for single-variable comparisons. Two-way ANOVA with repeated measures was used for the paw-edema time course data (0.5 to 6 h), with treatment as the between-subjects factor and time as the within-subjects factor. Two-way ANOVA was used to assess interactions between multiple experimental factors where applicable. Multiple comparison testing was conducted using Dunnett’s post hoc method to compare treatment groups against relevant controls. The threshold for statistical significance was established at *p* = 0.05, with results denoted as follows: **** *p* < 0.0001, *** *p* < 0.001, ** *p* < 0.01, and * *p* < 0.05.

## 5. Conclusions

This study establishes that *Gelidiopsis variabilis* polysaccharides, particularly GV-1A and GV-2A, function as immunometabolic modulators that are associated with inflammation resolution in this model system. Mechanistically, they were associated with arginine accumulation and complete elimination of the TLR4 agonist myristate (14:0), which is consistent with a pro-resolution metabolic phenotype. However, direct pathway measurements are required to confirm the involvement of TLR4/NF-κB signaling. *In vitro* GV fractions upregulate resolution markers (*IL-10*, *Arg1*, HO-1) and outperformed meloxicam in reducing edema. These findings are consistent with a new paradigm for algal polysaccharide bioactivity, wherein efficacy may be associated not solely by receptor binding but also with the ability to modulate macrophage metabolism toward inflammation resolution. GV polysaccharides therefore represent promising candidates for developing future resolution-based therapeutics for inflammatory diseases.

## Figures and Tables

**Table 1:** Characteristic proton and carbon NMR chemical shifts from GV polysaccharides for fraction GV-2A.

**^1^** **H NMR**	**H1**	**H2**	**H3**	**H4**	**H5**	**H6**
β-d-galactose (G)	4.58	3.63	3.76	4.15	3.72	3.78
6-methylated β-d-galactose (G6M)	-	4.30	3.80	4.20	4.20	4.20
Galactose-6-sulfate (G6S)	4.46	4.38	3.76	4.17	-	4.21
3,6-anhydro-α-*l*-galactose (LA)	5.12	4.12	4.55	4.69	4.57	4.02
**^13^** **C NMR**	**C1**	**C2**	**C3**	**C4**	**C5**	**C6**
β-d-galactose (G)	104.76	72.33	84.40	70.60	77.60	63.83
6-methylated β-d-galactose (G6M)	-	72.28	82.05	71.05	71.05	69.62
Galactose-6-sulfate (G6S)	106.01	72.35	83.33	71.27	-	69.65
3,6-anhydro-α-*l*-galactose (LA)	100.80	71.15	82.3	79.60	77.60	71.40

**Table 2:** Macrophage-specific fatty acid remodeling by *G. variabilis* fraction GV-1A and 2A, and correlation with bioactivity. Arrow symbol: ↑, increased.

Cell Line and Primary Function	Key Fatty Acid Alterations (GV-1A/GV-2A vs. Control)	Inferred Metabolic State/Mechanism	Correlation with Observed Bioactivity
RAW264.7— immune regulation and resolution	Reduced levels of myristate (14:0) and pentadecanoate (15:0) ↑ Longer-chain FAs (16:0, 16:1) Isomer shift and ↑ *trans*-18:1 (elaidate)	- Removal of a potent TLR4 agonist (myristate) and metabolic rewiring away from a pro-inflammatory lipid milieu. - Suggests a shift towards an oxidative, anti-inflammatory (M2-like) phenotype [34,35].	- Provides metabolic evidence consistent with the *in vivo* efficacy. - Offers a potential metabolic explanation for resolution- associated anti-inflammatory activity. - Correlates with M2-like polarization (↑ Arg1, IL-10, TGF-β1) and superior performance versus meloxicam.

**Table 3:** Comparative structural, metabolic, and functional profiles of *G. variabilis* polysaccharides derived from cold vs. hot extraction. Arrow symbols: ↑, increased; ↓, decreased.

Aspect	Feature	Cold Extraction (GV-1A)	Hot Extraction (GV-2A, GV-2B, GV-3A)
Molecular properties	MW range	360 kDa	1480–3006 kDa
	PDI	~24.8 (broad distribution)	~6.3–14.8 (more homogeneous)
	Sulfate content	6.60 ± 0.05%	16.66–21.53%
	Key structural motifs	κ-carrageenan-like; high 3,6-anhydro-α-L-galactose (LA)	Hybrid κ/β-carrageenan; methoxylated/sulfated galactans
Metabolic reprogramming	Amino acid shifts	↑ Arginine accumulation (4.61% to 7.06%)	↑ Arginine accumulation (variable intensity)
	Fatty acid remodeling	Complete elimination of myristate (14:0) and pentadecanoate (15:0); ↑ elaidate (trans-18:1)	Variable shifts; preservation of MUFA pools
Immunological responses	Immune modulation	Potent anti-inflammatory macrophage polarization (*Arg1* ↑, iNOS ↓); strong phagocytic activation (138–149%)	Immunostimulatory (NO production 27–37 μM) with later anti-inflammatory resolution
Key bioactive outcomes	*In vivo* efficacy (paw edema)	Dose-dependent edema reduction (66% inhibition at 400 mg/kg); superior to meloxicam in upregulating *IL-10*, *TGF-β1*, *Arg1*, HO-1	Effective edema reduction (up to 60% inhibition); strong upregulation of resolution markers
Mechanism	Proposed mechanism of action	Metabolic reprogramming toward anti-inflammatory macrophage polarization via arginine accumulation and myristate elimination	Immunomodulation via TLR/NF-κB modulation; metabolic support for immune resolution
Application	Potential applications	Systemic anti-inflammatory therapy; immunometabolic interventions	Localized immunomodulatory therapies

## Data Availability

The data presented in this study are available on request from the first author, and the data sets and materials are contained within the paper.

## Supplementary Materials

The following supporting information can be downloaded: https://doi.org/10.17632/bz2fnzmxhm.1 (access date: 19 August 2026), and https://doi.org/10.5281/zenodo.22012169 (access date: 19 August 2026).

## References

[1] Ciancia M., Matulewicz M.C., Tuvikene R. (2020). Structural diversity in galactans from red seaweeds and its influence on rheological properties. Front. Plant Sci..

[2] Chumsook K., Praiboon J., Fu X. (2023). Sulfated galactans from agarophytes: Review of extraction methods, structural features, and biological activities. Biomolecules.

[3] Tuvikene R., Robal M., Fujita D., Saluri K., Truus K., Tashiro Y., Ogawa H., Matsukawa S. (2015). Funorans from *Gloiopeltis* species. Part I. Extraction and structural characteristics. Food Hydrocoll..

[4] Atya M.E., Yang X., Tashiro Y., Tuvikene R., Matsukawa S. (2023). Structural and physicochemical characterization of funoran extracted from *Gloiopeltis furcata* by different methods. Algal Res..

[5] Kraithong S., Bunyameen N., Jaisan C., Liu Y., Sangsawad P., Huang R., Shi X. (2025). Gelling, thickening, and biological properties of marine algal polysaccharides: Implications for food applications. Carbohydr. Polym..

[6] Besednova N.N., Zaporozhets T.S., Kuznetsova T.A., Makarenkova I.D., Kryzhanovsky S.P., Fedyanina L.N., Ermakova S.P. (2020). Extracts and marine algae polysaccharides in therapy and prevention of inflammatory diseases of the intestine. Mar. Drugs.

[7] Premarathna A.D., Ahmed T.A., Kulshreshtha G., Humayun S., Darko C.N.S., Rjabovs V., Hammami R., Critchley A.T., Tuvikene R., Hincke M.T. (2024). Polysaccharides from red seaweeds: Effect of extraction methods on physicochemical characteristics and antioxidant activities. Food Hydrocoll..

[8] Generalov E., Yakovenko L. (2023). Receptor basis of biological activity of polysaccharides. Biophys. Rev..

[9] Taraballi F., Corradetti B. (2026). Sugar-coated immunometabolism: Functionalized biomaterials as metabolic regulators of immune responses. J. Mater. Chem. B.

[10] Hu X., Goff H.D. (2018). Fractionation of polysaccharides by gradient non-solvent precipitation: A review. Trends Food Sci. Technol..

[11] Gieroba B., Kalisz G., Krysa M., Khalavka M., Przekora A. (2023). Application of vibrational spectroscopic techniques in the study of the natural polysaccharides and their cross-linking process. Int. J. Mol. Sci..

[12] Hentati F., Tounsi L., Pierre G., Barkallah M., Ursu A.V., Ben Hlima H., Desbrières J., Le Cerf D., Fendri I., Michaud P. (2022). Structural characterization and rheological and antioxidant properties of novel polysaccharide from calcareous red seaweed. Mar. Drugs.

[13] Arata P.X., Fernández P.V., Ciancia M. (2015). Determination of Substitution Patterns of Galactans from Green Seaweeds of the Bryopsidales. Natural Products From Marine Algae: Methods and Protocols.

[14] Trivedi T.J., Srivastava D.N., Rogers R.D., Kumar A. (2012). Agarose processing in protic and mixed protic–aprotic ionic liquids: Dissolution, regeneration and high conductivity, high strength ionogels. Green Chem..

[15] Alencar P.O.C., Lima G.C., Barros F.C.N., Costa L.E., Ribeiro C.V.P., Sousa W.M., Sombra V.G., Abreu C.M.W., Abreu E.S., Pontes E.O. (2019). A novel antioxidant sulfated polysaccharide from the algae *Gracilaria caudata*: In vitro and in vivo activities. Food Hydrocoll..

[16] Van de Velde F., Knutsen S.H., Usov A.I., Rollema H.S., Cerezo A.S. (2002). 1H and 13C high resolution NMR spectroscopy of carrageenans: Application in research and industry. Trends Food Sci. Technol..

[17] Nagahawatta D.P., Liyanage N.M., Jayawardena T.U., Yang F., Jayawardena H.H.A.C.K., Kurera M.J.M.S., Wang F., Fu X., Jeon Y.J. (2023). Functions and values of sulfated polysaccharides from seaweed. Algae.

[18] Wei Z., Huang Q. (2019). Assembly of protein–polysaccharide complexes for delivery of bioactive ingredients: A perspective paper. J. Agric. Food Chem..

[19] Premarathna A.D., Ahmed T.A., Rjabovs V., Hammami R., Critchley A.T., Tuvikene R., Hincke M.T. (2024). Immunomodulation by xylan and carrageenan-type polysaccharides from red seaweeds: Anti-inflammatory, wound healing, cytoprotective, and anticoagulant activities. Int. J. Biol. Macromol..

[20] Santos S.A., Félix R., Pais A.C., Rocha S.M., Silvestre A.J. (2019). The quest for phenolic compounds from macroalgae: A review of extraction and identification methodologies. Biomolecules.

[21] Premarathna A.D., Sooäär A., Ahmed T.A., Rjabovs V., Hincke M.T., Tuvikene R. (2024). Isolation, structural characterization and biological activities of polysaccharides from *Chondrus crispus*. Food Hydrocoll..

[22] Can B., Sanlier N. (2026). Secret heroes of the sea: Brown macroalgae and their bioactive powers—A narrative review. Front. Nutr..

[23] Wang Y., Luo J., Xu C., Hu D., Li Y., Ye Y., Yang J., Chen X., Li C., Zhu K. (2026). Recent Advance in Marine Polysaccharides: Structure, Anti-Inflammatory Mechanisms, and Functional Applications. Mar. Drugs.

[24] Savic S., Petrovic S., Knezevic-Jugovic Z. (2026). Separation Strategies for Polyphenols from Plant Extracts: Advances, Challenges, and Applications. Separations.

[25] Rodríguez-Valentín M., López S., Rivera M., Ríos-Olivares E., Cubano L., Boukli N.M. (2018). Naturally derived Anti-HIV polysaccharide peptide (PSP) triggers a Toll-like receptor 4-dependent antiviral immune response. J. Immunol. Res..

[26] Bogdan C. (2001). Nitric oxide and the immune response. Nat. Immunol..

[27] Fujiwara N., Kobayashi K. (2005). Macrophages in inflammation. Curr. Drug Targets-Inflamm. Allergy.

[28] Strzelec M., Detka J., Mieszczak P., Sobocińska M.K., Majka M. (2023). Immunomodulation—A general review of the current state-of-the-art and new therapeutic strategies for targeting the immune system. Front. Immunol..

[29] Thapa B., Lee K. (2019). Metabolic influence on macrophage polarization and pathogenesis. BMB Rep..

[30] Rath M., Müller I., Kropf P., Closs E.I., Munder M. (2014). Metabolism via arginase or nitric oxide synthase: Two competing arginine pathways in macrophages. Front. Immunol..

[31] Orecchioni M., Ghosheh Y., Pramod A.B., Ley K. (2019). Macrophage polarization: Different gene signatures in M1(LPS+) vs. classically and M2(LPS–) vs. alternatively activated macrophages. Front. Immunol..

[32] Viola A., Munari F., Sánchez-Rodríguez R., Scolaro T., Castegna A. (2019). The metabolic signature of macrophage responses. Front. Immunol..

[33] Galván-Peña S., O’Neill L.A. (2014). Metabolic reprograming in macrophage polarization. Front. Immunol..

[34] Lancaster G.I., Langley K.G., Berglund N.A., Kammoun H.L., Reibe S., Estevez E., Weir J., Mellett N.A., Pernes G., Conway J.R.W. (2018). Evidence that TLR4 is not a receptor for saturated fatty acids but mediates lipid-induced inflammation by reprogramming macrophage metabolism. Cell Metab..

[35] Kirkham P. (2007). Oxidative stress and macrophage function: A failure to resolve the inflammatory response. Biochem. Soc. Trans..

[36] Tsai Y.W., Lu C.H., Chang R.C.A., Hsu Y.P., Ho L.T., Shih K.C. (2021). Palmitoleic acid ameliorates palmitic acid-induced proinflammation in J774A. 1 macrophages via TLR4-dependent and TNF-α-independent signallings. Prostaglandins Leukot. Essent. Fat. Acids.

[37] Wu N., Wang P., Ran X., Li L., She Y., Wang J., Zeng D., Li X., Jia Y., Huang Y. (2025). Palmitic acid induces macrophage sialylation via TLR4/MyD88/TRAF6/NF-κB signaling. Biochim. Biophys. Acta (BBA)-Mol. Basis Dis..

[38] Hou N., Zhou H., Li J., Xiong X., Deng H., Xiong S. (2024). Macrophage polarization and metabolic reprogramming in abdominal aortic aneurysm. Immun. Inflamm. Dis..

[39] Wang B., Dai T., Sun W., Wei Y., Ren J., Zhang L., Zhang M., Zhou F. (2021). Protein N-myristoylation: Functions and mechanisms in control of innate immunity. Cell. Mol. Immunol..

[40] Morvaridzadeh M., Alami M., Berrougui H., Boumezough K., Sidibé H., Salih I., Sadki K., Khalil A. (2025). Polyphenol-Microbiota Interactions in Atherosclerosis: The Role of Hydroxytyrosol and Tyrosol in Modulating Inflammation and Oxidative Stress. Nutrients.

[41] Wahnou H., Chgari O., Ndayambaje M., Hba S., Ouadghiri Z., Limami Y., Oudghiri M. (2025). Carrageenan and TLR4 Crosstalk: A Comprehensive Review of Inflammatory Responses in Animal Models. Recent Adv. Inflamm. Allergy Drug Discov..

[42] Davran Bulut S., Yaktubay Döndaş N., Koçhan S., Arslan B.N., Tamer M.A., Osmani M., Baraketi S., Khwaldia K., Zhang Z., Döndaş H.A. (2026). Marine Bioactive Compounds from Functional Seafoods: Pharmacological Mechanisms and Health Applications. Mar. Drugs.

[43] Wang S., Ni L., Fu X., Duan D., Xu J., Gao X. (2020). A sulfated polysaccharide from Saccharina japonica suppresses LPS-induced inflammation both in a macrophage cell model via blocking MAPK/NF-κB signal pathways in vitro and a zebrafish model of embryos and larvae in vivo. Mar. Drugs.

[44] Couper K.N., Blount D.G., Riley E.M. (2008). IL-10: The master regulator of immunity to infection. J. Immunol..

[45] Saraiva M., O’Garra A. (2010). The regulation of IL-10 production by immune cells. Nat. Rev. Immunol..

[46] Wynn T.A. (2004). Fibrotic disease and the Th1/Th2 paradigm. Nat. Rev. Immunol..

[47] Borthwick L.A., Wynn T.A., Fisher A.J. (2013). Cytokine mediated tissue fibrosis. Biochim. Biophys. Acta (BBA)-Mol. Basis Dis..

[48] Gordon S., Martinez F.O. (2010). Alternative activation of macrophages: Mechanism and functions. Immunity.

[49] Murray P.J., Wynn T.A. (2011). Protective and pathogenic functions of macrophage subsets. Nat. Rev. Immunol..

[50] Ryter S.W., Alam J., Choi A.M. (2006). Heme oxygenase-1/carbon monoxide: From basic science to therapeutic applications. Physiol. Rev..

[51] Immenschuh S., Ramadori G. (2000). Gene regulation of heme oxygenase-1 as a therapeutic target. Biochem. Pharmacol..

[52] Serhan C.N., Savill J. (2005). Resolution of inflammation: The beginning programs the end. Nat. Immunol..

[53] Perretti M., D’Acquisto F. (2009). Annexin A1 and glucocorticoids as effectors of the resolution of inflammation. Nat. Rev. Immunol..

[54] Arlov Ø., Rütsche D., Asadi Korayem M., Öztürk E., Zenobi-Wong M. (2021). Engineered sulfated polysaccharides for biomedical applications. Adv. Funct. Mater..

[55] Yuan D., Li C., Huang Q., Fu X., Dong H. (2023). Current advances in the anti-inflammatory effects and mechanisms of natural polysaccharides. Crit. Rev. Food Sci. Nutr..

[56] Moresco E.M.Y., LaVine D., Beutler B. (2011). Toll-like receptors. Curr. Biol..

[57] Ang Y., Ng A.J., Liao W., Wong K.K., Wong W.F., Peh H.Y. (2025). Resolution of Innate Immune Cells with Pro-Resolving Lipid Mediators in Idiopathic Pulmonary Fibrosis. J. Leukoc. Biol..

[58] Berezhnaya Y.D., Kazachenko A.S., Kazachenko A.S., Malyar Y.N., Borovkova V.S. (2024). Sulfation of various polysaccharide structures: Different methods and perspectives. Chemistry.

[59] Dubois M., Gilles K., Hamilton J., Rebers P., Smith F. (1951). A colorimetric method for the determination of sugars. Nature.

[60] Tabatabai M.A. (1974). A rapid method for determination of sulfate in water samples. Environ. Lett..

[61] Box J.D. (1983). Investigation of the Folin-Ciocalteau phenol reagent for the determination of polyphenolic substances in natural waters. Water Res..

[62] Premarathna A.D., Robal M., Bai R.G., Ahmed T.A., Rjabovs V., Hincke M.T., Tuvikene R. (2025). Bioactivities of *Nostoc* sp. polysaccharides: Anti-inflammatory, wound healing, cytoprotective, and anticoagulant effects. Int. J. Biol. Macromol..

[63] Premarathna A.D., Ranahewa T.H., Wijesekera S.K., Harishchandra D.L., Karunathilake K.J.K., Waduge R.N., Wijesundara R.R.M.K.K., Jayasooriya A.P., Wijewardana V., Rajapakse R.P.V.J. (2020). Preliminary screening of the aqueous extracts of twenty-three different seaweed species in Sri Lanka with in-vitro and in-vivo assays. Heliyon.

[64] Shen Y., Xu J., Zhi S., Wu W., Chen Y., Zhang Q., Zhou Y., Deng Z., Li W. (2022). MIP from legionella pneumophila influences the phagocytosis and chemotaxis of RAW264.7 macrophages by regulating the lncRNA GAS5/miR-21/SOCS6 axis. Front. Cell. Infect. Microbiol..

[65] Alttoa A., Eller M., Herm L., Rinken A., Harro J. (2007). Amphetamine-induced locomotion, behavioral sensitization to amphetamine, and striatal D2 receptor function in rats with high or low spontaneous exploratory activity: Differences in the role of locus coeruleus. Brain Res..

[66] Oldekop M.L., Herodes K., Rebane R. (2014). Study of the matrix effects and sample dilution influence on the LC–ESI–MS/MS analysis using four derivatization reagents. J. Chromatogr. B.

[67] Premarathna A.D., Dziubinska-Kuehn K.M., Sooäär A., Pushpitha N.P., Darko C.N.S., Bai R.G., Reile I., Tuvikene R. (2026). Sulfated galactans from *Gracilaria corticata* reprogram metabolism, activate Immunity, promote regeneration, and exhibit anti-melanoma activity. iScience.

[68] Winter C.A., Risley E.A., Nuss G.W. (1962). Carrageenin-induced edema in hind paw of the rat as an assay for antiinflammatory drugs. Proc. Soc. Exp. Biol. Med..

[69] Vinegar R., Schreiber W., Hugo R. (1969). Biphasic development of carrageenin edema in rats. J. Pharmacol. Exp. Ther..

[70] Livak K.J., Schmittgen T.D. (2001). Analysis of relative gene expression data using real-time quantitative PCR and the 2^−ΔΔCT^ method. Methods.

[71] Bustin S.A., Benes V., Garson J.A., Hellemans J., Huggett J., Kubista M., Mueller R., Nolan T., Pfaffl M.W., Shipley G.L. (2009). The MIQE Guidelines: M inimum I nformation for Publication of Q uantitative Real-Time PCR Experiments. Clin. Chem..

